# Clinical Review, and Hospital Reports

**Published:** 1841-07-01

**Authors:** 


### Clinical Review.

### GUY'S HOSPITAL.

Guy's Hospital Reports, No. XII. April, 1841. Edited by George H. Barlow, M.A. &c. &c. and James P. Babington, M.A. &c. &c.

The present number contains the following articles :—Observations on Epilepsy; read to the Physical Society of Guy's Hospital, 1841, by B. G. Babington, M.D. F.R.S.

On Poisoning by Arsenic; the quantity required to destroy life; by Alfred S. Taylor.

Some further Illustrations of the Safety-Valve Function of the Heart; by T. Wilkinson King, (with plate.)

Statistical Report of Guy's Hospital Lying-in Charity, from October, 1833, to October, 1840; with Cases and Observations; by John C. W. Lever, F.S.S.

Report on the Value of Electricity, as a Remedial Agent in the Treatment of Diseases; by Golding Bird, M.D. A.M. F.L.S. &c.

Observations on real and supposed Pathological Conditions of the Urine; by G. O. Rees, M.D.

Some Observations on Stricture of the Urethra, Catheterism, and False Passage; founded on post-mortem inspection; by Edw. Cock.

Cursory Observations on some Cerebral Affections of Children; read before the Physical Society of Guy's Hospital; by H. Marshall Hughes, M.D.

On the Existence of Arsenic as a Natural Constituent of Human Bones; by G. O. Rees, M.D.

Observations on the Absorption of Metals into the Blood, in Cases of Poisoning; illustrated by an account of a case of poisoning by lead, occurring in a cow under the care of Mr. Cherry; by Alfred S. Taylor.

Account of a Fœtus in Utero, invested by False Membrane; by Henry Oldham, (with plate).

Cases of Incised Wound of the Knee-joint, of Fluid in the Thyroid Body, and of Abnormal Thymus Gland. Communicated by Sir Astley Cooper, Bart. with plates.

Miscellaneous Cases :—Case 1. Bone passed from the Urethra. Communicated by Mr. Bransby Cooper.—Case 2. Deficiency of the Pectoral Muscles. Reported by Mr. Alfred Poland, (with plate.)—Case 3. Sequel of a Case of Stricture of the Urethra; by Bransby B. Cooper, F.R.S.—Case 4. Case of Fæcal Abscess. Reported by Mr. Caleb Taylor, (with plate.)—Case 5. Ulceration of the Stomach, terminating Life by Hæmorrhage. From the Note Book of the late Mr. Bryant, (with plate.)

Case of a remarkable Enlargement of the Female Breast; by S. Ashwell, M.D. (with plate.)

Observations on the Laws which regulate the Deposition of Tubercles; with Practical Inferences applicable to the Prophylactic Treatment of Phthisis; by G. H. Barlow, M.A. and L.M.

History of the last Illness of Sir Astley Cooper, Bart.; and Examination of the Body after Death.

We shall run over these papers in succession, and select the principal points in them for notice.

### I. Observations on Epilepsy. By B. G. Babington, M.D. F.R.S.

It is the object of this paper to give some illustrations of epilepsy, and to offer some reasons for thinking that it depends on a functional, and not a structural change; and some grounds for the belief, that in many instances it admits of cure. Dr. Babington is of opinion that women are more frequently affected than men. He thus alludes to the occurrence of epilepsy in the lower animals.

"'The meagrims, sturdy, or turnsick,' says Blaine, 'may be considered as a species of epilepsy, to which horses are not unfrequently subject; and in which, without previous notice, the animal, if in exercise, stops short, shakes the head, looks irresolute and wandering; in which state he continues a few minutes, and then proceeds as before. In more violent cases, he falls at once to the ground; or first runs round, and then sinks senseless. The whole system appears agitated by strong convulsions; the horse dungs and stales insensibly; is at some times violent, and at others more passive, but is equally unconscious of every thing around, in both. After remaining a longer or shorter period in this way, his faculties return, and he rises.'—3d Edition, p. 471. Dogs and cats are also subject to a loss of consciousness, under which they fall down and struggle; and caged birds are often similarly affected."

Dr. Babington relates a case in which there were first distinct epileptic seizures, and afterwards paroxysms of mere temporary loss of consciousness, unattended with falling or with convulsions. He considers these allied to the former. He adds—"Such I hold to be the nature of the case in a lady of my acquaintance, 73 years of age, who for the last two years has been observed occasionally to lose consciousness for a few seconds while reading or speaking; so that she will stop suddenly in the midst of a sentence, as if her attention had been accidentally arrested by some external circumstance. After this pause, she feels confused; and at length, becoming collected, will proceed with her sentence as if nothing had interrupted her. She evidently desires, however, to conceal what has occurred, and is annoyed if any notice is taken of the circumstance. What is commonly called absence of mind seems to be somewhat allied to this state, although a still slighter aberration of intellect: and with persons who are subject to this affection of the sensorium, it will sometimes happen, that in the midst of a narrative they will suddenly forget the thread of their discourse, and a degree of obliviousness or confusion will for a moment ensue, which bears a near resemblance to the slight epileptic seizures which I have been describing." Perhaps it is rather too hard on absent people to look on them as little better than epileptics. Absence of mind very frequently, nay most frequently, depends on its over-occupancy with a particular train of ideas, rather than on the interruption of consciousness. The attacks of semi-insensibility have been described as epileptic in their character by others. Esquirol, for instance, alludes to them.

Dr. Babington relates a good many cases which we do not think it necessary to insert. They illustrate the principal and too familiar features of epilepsy. He remarks, in reference to the aûra epileptica—it is not necessarily connected with epilepsy at all; being sometimes felt by those who suffer from cardialgia, pyrosis, and other symptoms of dyspepsia. A near relative of my own furnishes an example. He is much subject to headaches, dependent on a disordered state of stomach; under which he ejects, by a kind of ruminating action hardly amounting to retching, large quantities of acid fluid. These attacks are often ushered in by a sensation of tingling in one arm, which mounts up from his fingers' ends, and gradually advances towards the face on the same side, affecting one half of the tongue, palate, and lips."

Dr. Babington compares epilepsy to sleep, to the torpor produced by mesmerism, and institutes several parallels of this sort. As a sample of his reasoning we may quote a brief passage.

"If an analogy be admitted between the phenomena of cramp and those of the suspension of consciousness in epilepsy, I might be led to suspect that both are dependent upon some change of position in the nervous molecules, having the effect of cutting off nervous influence; and, that so long as this change of position continues, they are either no longer capable of discharging at all, or cannot discharge in an orderly and normal manner the functions which they are destined to perform."

Our author's notion of the pathological nature of the malady will be seen not to differ materially from that entertained by most well-informed men.

"Post-mortem examination, where it exhibits any appreciable deviation from health, leads to the same conclusion. All kinds of lesions, of the bones of the cranium, whether from accidents or original malformation, of the membranes of the brain, of the substance of the brain, of the position of the brain by adventitious growths or hydatids, of the medulla oblongata, of the spinal marrow, and of various remote organs, have been found in connexion with epilepsy: yet all of such various kinds, as to lead to a belief that it can only be one and the same specific effect on the nervous system, which is remotely and secondarily induced by each. That specific effect, for want of a more precise notion of it, I must call irritation; and as even various kinds of mental emotions are capable of producing it, I am here again led to the conclusion, that it is not in its essence an organic change, but merely some temporary alteration in the arrangement or position of particles."

With Dr. Babington's methodus *medendi*(?) we may conclude.

"Where the irritation, which I suppose to be the proximate cause of epilepsy, is complicated with and remotely dependent on organic change of structure, we cannot of course expect that treatment, of whatever kind, will either effect a cure of the disease, or throw any light upon its cause. Again: where it is combined with a state of plethora—never occurring but when the person is in a horizontal position, or after a full meal, and attacking those whose habit is gross and circulation forcible,—means, the opposite of those which would be adapted to remove the proximate cause, may be needed. Bloodletting may be necessary, as well as active evacuants, and an antiphlogistic line of treatment; but, with these exceptions, it is my belief that the same class of remedies which is best calculated to give tone to the system under tic doloureux, and other spasmodic states of the nerves of motion and sensation dependent on nervous irritation, is also most applicable to this disease; and that the various preparations of bark, of iron, of arsenic, of silver, or of zinc, are those from the administration of which we shall derive the greatest advantage. Of the sulphate of zinc, I have lately made more frequent use than of any of the rest; and as it has proved efficacious in some cases, I shall conclude by an allusion to one or two of the most striking. Like all other remedies, it will often fail; but whether useful or not, it has the merit of being a safe remedy, and that when continued for a longer period, and in very much larger doses than is usually supposed. Those large doses seem sometimes, though by no means always, essential to its efficacy; and the method of reaching them, without producing nausea, is by a gradual increase, as in the case of emetic tartar. Thus Frances D—— (page 5), to whom I have more than once alluded, was enabled to take thirty-six grains, three times a day, for several weeks, without its producing any sickness or other untoward effect. This, in her case, was the maximum tonic dose; for when she took forty-two grains, which she continued to do for one week, she lost her appetite, and felt much sickness."

### II. On Poisoning by Arsenic. The Quantity required to destroy Life. By Alfred S. Taylor.

Mr. Taylor shews that much difficulty exists in determining the minimum dose of arsenic, capable of destroying life. A witness is often asked the question.

Dr. Smith states, in his Analysis of Medical Evidence, that on a trial which took place at York in 1816, the medical witness, in reply to a question of this kind, said, that *sixty* grains of arsenic would produce violent vomiting; that two drachms, in his opinion, would be sufficient to destroy life; although it would require at least *one ounce and a half* of that which is commonly sold in the shops. The evidence of this witness must have been very erroneously reported: or he must have been ill informed on the subject on which his opinion was asked. As some extenuation, however, of this unfortunate specimen of medical evidence, it is proper to observe, that it was very much the practice, formerly, to adulterate white arsenic with plaster of Paris, or other innocent powders. It is not unlikely that this practice may still continue in some parts of the country.

Mr. Taylor, however, has not found any impurity of this description in specimens of arsenic obtained from various London chemists.

According to Hahnemann, *two grains* of arsenic might suffice to destroy life in the course of a few days; but he adduces no case in support of this opinion. Dr. Christison, in his Treatise on Poisons (2d ed. 1832), states, that the smallest dose of the solid poison, which he has read of, amounted to *thirty* grains of the powder. This dose proved fatal in six days. The smallest actually fatal dose which he has found recorded, was four grains and a half; but in this case, the subject was a child four years old; death took place in six hours. In this instance, the poison was taken in solution.

Mr. Taylor thinks it certain that a much smaller quantity than thirty grains of this poison would destroy life. Our means of getting at the fact is to shew what are the largest quantities that have been taken without proving fatal, excluding those instances in which a large quantity of arsenic has been swallowed, in some bulky menstruum, or on a full stomach, owing to which early and violent vomitting has sometimes ensued, and the parties have ultimately recovered.

"Again, in considering this question, it is necessary to determine, whether age, sex, peculiarity of constitution, or the state of bodily health, may not influence the operation of this poison. In respect to *age*, it appears to be established by many cases, that arsenic more powerfully affects an infant that an adult; and it is not improbable that an aged and infirm person might be killed by a dose from which a younger person would recover. In respect to the influence of *sex*, I am not aware of any facts from which a definite opinion can be drawn, or, indeed, that attention has been directed to this point: it is however certain, that fatal cases of suicidal poisoning by arsenic are much more common among females than males.* That arsenic affects some *constitutions* more than others, appears to be well established from its employment in medicine. One patient is observed to be seriously affected by a dose which scarcely has any influence on another; and there are persons to whom arsenic cannot be administered at all. In making an estimate of the quantity requisite to destroy life, this point must not be omitted; for although the poison may be in comparatively small doses, fatal to all constitutions, we have here an explanation of the fact, that, *cæteris paribus*, from the exhibition of the same dose, one person may die and the other recover. Lastly, with regard to the state of *bodily health*;—we cannot refuse our assent to the view, that when the body is weakened by disease, it is possible that this active poison, in a certain dose, would operate fatally on one who might have recovered from its effects had he been in ordinary health. We are, I think, in the absence of facts, entitled to draw this inference, from the general principle, that a diseased state of body is favourable to the action of most noxious agents."

Mr. Taylor points out the fallacy which may attend conclusions drawn from experiments on animals. He mentions an instance. In a case tried at Leicester

* This is probably owing to the fact, that suicide by poison is much more common among females. Males generally destroy themselves by cutting the throat, hanging, drowning, or by fire-arms.

some years ago, several medical witnesses gave strong evidence against a prisoner charged with poisoning a female. From the rapidly fatal effects of the poison (hydrocyanic acid) on dogs, they inferred that this must have been a case of murder, and not, as it was alleged in defence, of suicide. Fortunately for the prisoner, this evidence against him was set aside by circumstances, which proved that the deceased must have designedly taken the poison herself. This case, with some others which might be adduced, establishes, that we are not justified in forming a positive opinion of the fatal doses of poisons in men, from experiments on animals, however numerous or well conducted they may be.

Dr. Lachèse, of Angers, has inferred that a smaller dose than two grains may prove fatal. Thirteen persons were poisoned by partaking of bread into which arsenic had been wilfully introduced. They all suffered from the usual symptoms of arsenical poisoning, but recovered in the course of a few days. This was thought to be a favourable case for estimating the effects of arsenic in a certain dose; and, accordingly, the bread was submitted to a quantitative analysis, by several eminent chemists. According to the opinions of some of these chemists, the proportion of arsenic was about half a grain to each pound of bread; and, on inquiry, it was found that those who had partaken of the bread could not have eaten less than a quarter of a pound, and therefore must have taken about *one-eighth* of a grain of arsenic.

This dose, according to Dr. Lachèse, has no other effect than that of inducing speedy vomiting;—it does not affect the mucous membrane of the stomach, nor does it become absorbed.

In the cases where from half a pound to a pound of bread had been eaten, *i. e.* where from *one-quarter* to *one-half* grain of arsenic had been swallowed, more decided symptoms of poisoning were manifested. The mucous membrane of the stomach was seriously affected;—the irritation was speedily propagated to the intestines;—thence followed pain in the abdomen, nausea, vomiting—the vomited matters holding sufficient arsenic to give rise to an acrid sensation in the œsophagus. The nervous system also began to be affected by the poison.

Some of these persons, not suspecting the cause of their illness, partook of the bread the following day; and the symptoms of arsenical poisoning appeared under an aggravated form, being accompanied by vertigo and great prostration of strength.

From these and other observations, Dr. Lachèse was led to infer that one-eighth of a grain of arsenic may seriously indispose a healthy adult,—that from one-quarter to one-half grain will give rise to all the symptoms of poisoning,—and that in the dose of from one to two grains, arsenic may cause death.

Mr. Taylor justly remarks:—Allowing that the chemical analysis in this case was correctly made, it is difficult to understand how the arsenic should have been so uniformly mixed with a hundred pounds of bread, as to justify the inferences drawn respecting the effects of particular doses. One part of the dough may have received more than another: whereas the conclusions are made on the assumption, that the poison was equally diffused, and that each person took a fractional portion of arsenic, according to the weight of bread consumed.

On the whole, it will be seen that there is considerable difficulty in admitting that the arsenic was thus equally diffused through the bread; and hence we cannot place much confidence in the conclusions.

Mr. Taylor mentions a very remarkable case which occurred in town in October, 1839.

A gentleman was dining with a party at the house of a friend. After dinner, the wine was passed round as usual; when, in the course of a short time, three of the persons present were seized with symptoms indicative of poisoning. The gentleman having observed that those only suffered who had partaken of port-wine, immediately suspected that the wine was poisoned. The whole of it was put back into the bottle, and sent to Mr. Taylor next day for chemical examination.

The wine was clear, of the usual colour and odour, and possessed all the characters of good port-wine. There was a small quantity of fine white sediment at the bottom of the bottle. Mr. Taylor suspected that the poison was arsenic, and such proved to be the case—that substance being dissolved in the wine, and the undissolved substance consisting of it nearly in a state of purity. The quantity in solution amounting to 28.8 gr. and the undissolved sediment consisting of about 60 grains.

The quantity of wine taken by each of the persons present at the dinner was pretty accurately ascertained.

1. A child, aged sixteen months, took about two teaspoonfuls, and therefore about two fluid drachms. This quantity of wine contained 0.3, or about one-third of a grain of arsenic. In about twenty minutes this child became sick, vomited severely for three hours, and then recovered. The child was the first affected: it did not complain of pain, and its sickness was supposed to be owing to some other cause.

It is remarkable—considering what appears to be well established, that arsenic severely affects young children—that this child should have suffered so little from so large a dose. Admitting that one teaspoonful of wine only had been taken, this would have contained one-sixth of a grain of arsenic; a quantity sufficient, we might *à priori* imagine, to produce the most alarming symptoms, if not death, in so young a subject.

2. A lady, aged 52, took a wine-glassful; excepting the two teaspoonfuls, which she gave to the child. In from thirty to forty minutes, she complained of a general feeling of uneasiness, for which she could not account; she suffered no pain, but vomited violently for four hours. She then recovered. This lady could not have taken less than *one grain and a half of arsenic.*

3. A gentleman, aged 40, drank a glass and a half of the wine. About twenty minutes afterwards, he complained of feeling unwell, and took some brandy. Vomiting came on, which lasted for three hours; but this gentleman did not entirely recover from the effects of the poison until after the lapse of several days. He could not have taken less than *two grains and a half* of arsenic.

"All these cases," observes Mr. Taylor, "agree, in the absence of pain: the only symptoms of arsenical poisoning which they exhibited, were, the violent vomiting, and prostration of strength. To what are we to ascribe the recovery of the two adults, from doses of arsenic which would commonly be set down as sufficient to kill? It will be observed, that in estimating the quantity of poison taken by these persons, I have only considered that which was actually dissolved in the wine. It is possible, with a freshly-decanted bottle, that some of the finely-levigated powder may have been mechanically suspended in the wine, and swallowed by them. Confining ourselves to the portion really dissolved, we learn, by these cases, that a dose of arsenic amounting from one grain and a half to two grains and a half may be taken by an adult, not only without destroying life, but without even exciting very formidable symptoms.

It does not appear reasonable to attribute this exemption to peculiarity of constitution; since this would be rather an evasion than an explanation of the difficulty. It must also be made applicable to all who suffered; the child as well as the adults. Perhaps a more correct view is, to attribute the escape of these persons to the fact, that the poison was taken on a full stomach, after a meal; and that, becoming mixed up with the half-digested food, it was speedily and effectually ejected by vomiting. The fact of exemption, under these circumstances, was long ago observed by Morgagni. This explanation would apply at least to the two most difficult cases; namely, to the two adults, who took so large a quantity of the poison."

After some further remarks, he adds—"It appears to me to be a subject for further inquiry, whether this poison may not be taken in a much larger dose than is commonly supposed, without destroying life. Can we say it is impossible that a person should recover after having taken five or ten grains? or would a witness be justified in swearing that such a quantity of this poison must necessarily destroy life? These questions should, I think, at present be answered in the negative. All that we are justified in saying, is, that, judging from the effects of smaller doses, these quantities would *probably* kill; but that nothing certain was known as to the quantity which would constitute the lowest fatal dose of the poison. If a witness answered the above questions affirmatively, a court of law would probably require good grounds for the affirmative answers, or at once reject them." And no such grounds appear to exist.

Another point touched on by Mr. Taylor is the source of the arsenic in the wine. It had been, he says, according to his information "procured, as usual, from a wine-merchant. Bottles of wine from the same stock had been previously consumed without giving rise to any symptoms of poisoning. In short, from all I could learn, this appears to have been a solitary bottle poisoned in the stock. There is something calculated to excite the most painful feeling, in the idea that wine should be by any accident produced, at a private table, containing upwards of eighty grains of arsenious acid: and we cannot but regard it as a most fortunate circumstance, that, out of ten or twelve persons present, not more than three should have partaken of this wine, and in so small a quantity. Three or four glasses of the wine, perhaps even less, would probably have destroyed the life of an adult.

It appeared that the poison could not have been placed in the wine after it had been decanted; and there was no doubt that the bottle had not been interfered with, since it was first corked, and sent by the wine-merchant. Suspicion was immediately directed to the possibility of shot having been left in the bottle, this being known to contain a portion of arsenic, used in the manufacture to give it hardness: but when the bottle was delivered to me, there were no shot, nor any traces indicative of shot having been left in it. The white sediment was quite free from the presence of lead. The wine, although acid, probably from tartaric acid, contained no traces of lead dissolved; and altogether it seemed unnecessary to pursue this inquiry further. He did examine into it, however, and seems to have ascertained that the arsenic present in dust shot probably weighs no more than $\frac{1}{200}$th the weight of the lead. As a single pellet weighs about two grains, this would give $\frac{1}{100}$th grain of arsenic for each. Thus, allowing this to be an approximative estimate of the largest quantity of the poisonous metal likely to be present, it would follow, that an ounce of dust-shot would contain, on the average, about *five grains* of arsenic, in the state of arseniate and arseniuret of lead. It will now be seen, from this calculation, that to have produced the quantity of arsenious acid present in the poisoned wine, it would have required the separation of arsenic from about eleven ounces of shot! But even admitting that the arsenic in each pellet was in the large proportion of $\frac{1}{50}$th grain—a very improbable supposition—it would have required at least *five ounces and a half* of shot to have produced the quantity of arsenious acid discovered in the bottle. It is not likely, that, under any circumstances, so large a quantity of shot should have been accidentally left in a bottle; nor is it likely that all the arsenic should be thus extracted from the shot, without the lead becoming at the same time separated, and the wine strongly impregnated with the metal. But in this case, no soluble or insoluble salt of lead had been produced."

Mr. Taylor introduced some shot in which arsenic had been ascertained to be present, into some port and some sherry wine. They were not contaminated. And it, therefore, seems unreasonable to anticipate danger from this source. The following observations are of the first importance.

"Enough has been said to shew that the wine, in the case related, could not have derived its impregnation with arsenic from shot left in the bottle; and this unfortunate accident remains to be accounted for in other ways. The only explanation which occurs to me, is, that the bottle must have been used for containing the poison previously to the wine having been put into it. Thus arsenic is largely used by shepherds, as a lotion to kill the fly in sheep; also by farriers and others, for veterinary purposes. It is also sold as a bug-poison. Bottles, thus used for containing the poison, are bought by pedlars; and when a sufficient number is accumulated, they are sold. It is in this way that poisoned bottles may pass into the hands of wine-merchants, without their being aware of it; and unless more than usual caution is employed in cleaning them, the wine put into them will become poisoned, and give rise to serious consequences. The rinsing of the bottles, or even allowing them to soak in water, will not remove the arsenic effectually; for this commonly exists in the interior, adhering to the sides or edges in the form of a hard crust. Bottles which have contained poisonous substances of this kind should always be destroyed. Public attention has not been sufficiently drawn to this subject, owing to a want of a vigorous system of medical police; but I have reason to believe, from attending to the numerous cases of poisoning which have occurred within the last ten years in England, that many cases of this kind have occurred, especially with stone bottles such as those which are used for the sale of ginger-beer, in the streets of London. The illness or death has been ascribed to cholera, or some imprudence on the part of the affected person, when the real cause was probably some irritant poison.

About the same time that the case, which I have described, occurred in London, one somewhat similar happened in France. A father and son were seized with severe vomiting, immediately after their supper. It was found, on examination, that the wine which they had drunk was poisoned with arsenic, and was the cause of the symptoms. The iron antidote was administered, and they both recovered the following day. Some other members of the family, who had drunk the wine, were seized with vomiting. A large quantity of arsenic was found at the bottom of the bottle. It was presumed that the two persons who had suffered most severely had taken more than sufficient to occasion death; and that they would probably have died, had it not been for the timely application of the antidote. There is but little doubt that the bottle had been used for an arsenical lotion.

An interesting case is related by Dr. Christison, where six persons suffered severely from taking arsenic in champagne, while dining at the house of a friend, a baronet in Roxburghshire. The quantity of arsenic calculated to have been taken by each adult amounted to one grain. They all recovered. In this case, the wine was traced to have been sent poisoned by arsenic from a wine-merchant:— no doubt accidentally, and probably from the bottle having been previously used for an arsenical wash.

The following case will corroborate the view that has been taken of this source of accidental poisoning.

In August 1836, four persons of a private family in Jersey were seized with alarming symptoms of poisoning, after a meal. One of them, a lady who had been in a declining state of health, speedily sank under the symptoms: the other three recovered. It was at first thought that hemlock had been put into the broth by mistake; but the medical attendant, finding that the symptoms were those of irritant poisoning, directed his attention to the liquids which had been used at the meal. It appeared that some perry, contained in a stone bottle, had been drunk; and it was then suspected that the glaze in the bottle might have impregnated that acid liquid with a poisonous dose of lead. The bottle was washed out but no sediment was obtained, and it was therefore broken by the medical practitioner. He then found, at the bottom of the bottle, a thick incrustation of an earthy looking substance, in quantity exceeding an ounce, which adhered so firmly to the bottle, that it was necessary to remove it with a sharp knife. This substance, on examination, turned out to be arsenious acid, without any admixture of lead. The symptoms in all, and the cause of death in one of the persons, were thus satisfactorily explained.

In the opinion of the examiner, the arsenical crust had been there for some years. No washing or concussion of the bottle would have sufficed to dislodge it; and owing to the bottle being opaque, its presence never could have been suspected.

The history of this unfortunate case was, that a quantity of stone bottles had been purchased at Liverpool, for the purpose of bottling the perry which had been supplied to the deceased and her friends; and from the appearance of this bottle, no doubt could exist that it had been, at some previous time, used as a receptacle for bug-poison.

Considering the whole of these facts, I am inclined to believe that this is the true solution of the cause of the wine having been poisoned in the case narrated in this paper; and that, except in the great caution used by respectable wine-merchants, the public have no protection against such accidents being of much more frequent occurrence than they are."

### III. Statistical Report of Guy's Hospital Lying-in Charity, from Oct. 1833, to Oct. 1840. With Cases and Observations. By John C. W. Lever, F.S.S.

The Lying-in Charity attached to Guy's Hospital, we are informed, was founded by the Treasurer, at the close of the Autumn of 1833, for the purpose of providing poor women with medical attendance and medicines at their own habitations, during their confinement. Females who live within a reasonable distance from the Hospital, and are fit objects of this Institution, may avail themselves of its benefits, by entering their names in a book kept at the office of the charity. The Institution is managed by a Physician Accoucheur, two Assistant Accoucheurs, and a Female Attendant, to whom all applications are to be made. Pupils who are desirous of attending women during their confinement enter their names and residences at the office; but those *only* who have attended lectures, and are considered competent, have cases consigned to their care. The patients are allotted to each pupil by the assistant accoucheurs; who, in the distribution, keep in mind the residences both of pupil and patients, and the experience of the former, with the probability of a difficult or complicated labour. The Regulations of the Institution require a pupil, as soon as he receives a letter, to call on his patient; and if unavoidably prevented from attending her, he must give immediate notice at the office of the charity. Pupils are not permitted to administer the secale cornutum, nor to use any obstetric instrument, without the sanction or presence of one of the assistant-accoucheurs, or of the physician accoucheur. The Regulations further require, that if a patient has been in labour for more than twelve hours after the discharge of the liquor amnii, if there be any preternatural presentation, or if any unusual or urgent symptoms supervene, the pupil in attendance must obtain the advice of one of the assistant accoucheurs. After a patient's confinement, she is to be seen daily during the first week, and as long after as her condition may require. The letters given to the patients have blank certificates at the bottom; which pupils are required to fill up after delivery, stating the sex of the child, whether alive or still-born, the day of birth, &c. Each pupil is required to give into the office a report of the labour, stating its nature, &c.; and if deemed requisite, he must furnish a daily account of her symptoms, treatment, &c. These Regulations have been most strictly observed since the establishment of the institution: and they have been found not only to ensure the greatest attention towards the patients, but also to the pupils ready advice and assistance, affording all the clinical benefits of a Lying-in Hospital.

From 1833 to 1840, 4666 women have been attended by the pupils attached to the Lying-in charity.

A table is given with the view of shewing the number of women confined in each month, in seven years, extending from 1833 to 1840. It appears that in May the greatest number of confinements have taken place, and in October the least. The months run in the following order :—

| | | |
|---|---|---|
| May | September | August |
| December | January | November |
| March | July | June |
| February | April | October. |

These results differ from those published by MM. Quetelet and Villermè. The order of births among the town and country population of the Low Countries, during the twelve years from 1815 to 1826, was as follows :—

| COUNTRY POPULATION. | TOWN POPULATION. |
|---|---|
| February | February |
| March | March |
| December | January |
| April | April |
| January | December |
| { October } | November |
| { November } | September |
| September | October |
| May | May |
| August | August |
| June | June |
| July. | July. |

From another table it seems that the greatest number of women have been attended in their first confinements, and that the number gradually lessens in amount as the repetitions of pregnancy increase; for which there are obvious reasons.

Table III. distinguishes the sex in 4432 children born alive. Of these 2318 were males—2114 were females. Table IV. distinguishes the sex in 263 still-born children. Of these 157 were males—106 were females.

From a careful examination of these tables, it will be seen, that the proportion of males to females born alive is as 52.3, to 47.6; and the proportion of males to females still-born is as 59.69 to 40.3.

By comparing the two tables, it will also be evident, that there is an excess of 7 per 100 in the males still-born, as contrasted with the males born alive; and a corresponding defect of 7 per 100 in the females still-born, as compared with the females born alive. It also appears that the ratio of children still-born to the children born alive is 5.6 per 100. Dr. Collins, in his admirable "Practical Treatise on Midwifery," states, that during his residence as Master at the Dublin Lying-in Hospital, out of 16.654 children born, 1121 were still-born: this will give a ratio of 6.7 per 100. Casper, in his "Uber die Sterblichkeit der Kinder in Berlin," gives the following Table :—

| Places. | Births, for one Still Birth. |
|---|---|
| Strasburgh | 11 |
| Hamburgh. | 15 |
| Dresden | 17 |
| Paris | 19 |
| Berlin | 20 |
| Vienna | 24 |
| London | 27 |
| Brunswick | 33 |
| Stockholm | 36 |

The mean of this table will be about 1 still birth to 22 births; differing from the result of the Berlin Registers, which, kept for a period of sixty years, gives 19.8 as the mean.

The results of the Registers of Amsterdam from 1821 to 1832 give the number of births at 7282, and the number of still births at 430; making the proportion 1 still birth to 16.9 births.

At Paris, the "Annuaires du Bureau des Longitudes" give the following results:—

| | | |
|---|---|---|
| From 1823 to 1832, | Births .. .. .. | 287,639 |
| | Still Births .. .. | 16,190; |

the proportion being 1 still birth to 17.7 births.

Table V. shews the varieties of labour in 263 cases of still-born children. And—

Table VI. shews the varieties of labour in 4666 women, attended between October, 1833, and October, 1840. We must content ourselves with a summary of them.

It appears, says the reporter, that 4290 labours were natural; being in the proportion of 91.9, or nearly 92 per 100. Of this number, in 4266 cases the vertex presented, or in the proportion of 99 in 100. The cases of face-presentations amount only to 24, or 1 in 179 cases.—The following is the comparison between Guy's Charity and other Institutions.

| | Proportion. |
|---|---|
| Guy's . . . . . . . . . . . . | 1 Case in 179. |
| Mme. Boivin . . . . . . . . | 1 . . 275. |
| Merriman . . . . . . . . . . | 1 . . 450. |
| Dublin Hospital . . . . . . . | 1 . . 504. |

Of the 4266 cases of vertex-presentation, in 103 the children were still-born; viz. in the proportion of 1 in 42, or 2.4 per 100. In six of the 24 face-presentations, the children were still-born; or 1 in 4, or 25 per 100: this mortality exceeds that of children born under the same presentation in the Dublin Lying-in Charity, which was 1 in 8, or 12 per 100.—The following Table will shew the duration of labour in the 24 face-presentations:—

| | Hours. | | | | | | | | | | | | |
|---|---|---|---|---|---|---|---|---|---|---|---|---|---|
| Duration . . . . . . . . . . . . . . | 2 | 5 | 7 | 8 | 9 | 10 | 12 | 13 | 14 | 15 | 18 | 21 | 29 |
| Number of Cases . . . . . . | 1 | 1 | 2 | 3 | 1 | 5 | 3 | 1 | 1 | 1 | 3 | 1 | 1 |

*Premature Labours.*—By referring to the Table, it will be seen that 55 labours are reported as premature: of this number, 6 were induced. Of the 49 labours that spontaneously occurred, 40 children were still born, or 81.6 per 100. The ratio of premature still births to the total is 15.2 per 100. In the Dublin Lying-in Hospital, the ratio was 26.2 per 100. Six cases of premature labour are reported as having been induced. Of this number, one woman was admitted as a patient of Mr. Key's, in Dorcas Ward, labouring under osteosarcoma of the knee, involving the lower half of the femur, and the heads of the tibia and fibula: her case is reported at length in Guy's Hospital Reports, Vol. I. p. 323. Labour was induced in the remaining five cases, to supersede the necessity of perforation; that operation having been resorted to on previous occasions, to accomplish delivery, the pelvic diameters being seriously abbreviated.

The following table will shew the method of operating; the length of time that elapsed from the period of induction to the commencement of pain; and the completion of the labour, the presentation, and the event to the child.

| No. | Method of Induction. | Hours before occurrence of Pain. | Hours before completion of Labour, after the operation. | Presentation. | Event to Child. |
|---|---|---|---|---|---|
| 1. | Puncture, & Tinct. Secale | 27½ hrs. | 50 hrs. | Nates . . | Still. |
| 2. | Puncture . . . . . . | 12 .. | 20 .. | Feet abd. anterior. | Still. |
| 3. | Puncture . . . . . . | 12 .. | 40 .. | Nates . . | Still. |
| 4. | Puncture . . . . . . | 132 .. | 138 .. | Vertex . . | Alive. |
| 5. | Separation of Membranes | 11 .. | 27½ .. | Vertex . . | Still. |
| 6. | Puncture . . . . . . | 2 .. | 7½ .. | Vertex . . | Still. |

From this table, it will be seen that only one child was born alive, and that under a vertex-presentation: and it is remarkable, that, in this case, labour-pains did not commence until the expiration of 132 hours; and did not terminate until the expiration of 138 hours, from the period of operation.

The following table will shew the results of the practice of other operators:—

| | | Children born alive. |
|---|---|---|
| Mr. Barlow . . . . . . . . | operated 16 times; and in 2, premature labour occurred spontaneously | 18 |
| Dr. Denman . . . . . . . . . . | in 12 Cases . . . | 7 |
| Mr. Marshall . . . . . . . . . . | .. 4 . . . . . . . | 1 |
| Dr. Merriman . . . . . . . . . | .. 33 . . . . . . . | 13 |
| Dr. Hamilton . . . . . . . . . | .. 45 . . . . . . . | 41 |
| Guy's Lying-in Charity . . . . . . | .. 6 . . . . . . . | 1 |

*Protracted Labours.*—In 62 women the labour was protracted, being in the proportion of 1.32 per 100. The chief causes which led to the protraction of labour, were, imperfect uterine action; rigidity of the soft parts; disproportion between the head of the child and the pelvis of the mother; firm and extensive ossification of the fœtal head not allowing of compression.

In 16 cases out of 4666, delivery was effected by the instrumentality of the secale cornutum, making a proportion of about one case in 292 deliveries. These cases were the most favourable that could possibly offer for the exhibition of this drug; in them the presentations were natural, the parts fully prepared to admit with safety the passage of the child, and nothing but uterine pains appeared to be wanting to complete the delivery. In these cases, the secale acted speedily and effectually. The preparations of the drug administered, were, the pulvis ergotæ, the tinct. ergotæ, and the ethereal tincture of ergot. Mr. Lever adds:—

Of this latter preparation I can speak with the greatest confidence, having upon several occasions tested its efficacy and value; and in fact, I may say, when I have exhibited it, it has never failed to accomplish the object I had in view. It was first prepared for me by my friend Dr. G. O. Rees; and his method of preparation was published by me in the Medical Gazette of April 4, 1840. The dose I exhibit is m. xxx. to m. xl. given in sugar. In 21 cases only was delivery effected by the embryospastic instruments; viz. 9 by the forceps, and 12 by the vectis; or about 1 in 222. In 25 cases, the operation of lessening the child's head was had recourse to, being in the proportion of 1 in 187 deliveries. If we add the number of women delivered by the vectis and forceps to the number delivered by the assistance of the perforator, we shall have 46 instrumental deliveries in 4666 cases, or in the proportion of one case in about 101 deliveries.

The following table presents the proportion of Instrumental Deliveries at other Institutions.

| | | | |
|---|---|---|---|
| Dresden, Dr. Carus | 1 | in every | 13 |
| Berlin, Dr. Kluge | 1 | ...... | 15 |
| Heidelberg, Professor Naëglè | 1 | ...... | 28 |
| Marburg, Dr. Siebold | 1 | ...... | 9 |
| Vienna, Dr. Boer | 1 | ...... | 96 |
| Paris, Madame Boivin | 1 | ...... | 183 |
| Dublin Lying-in Hospital, Dr. Collins | 1 | ...... | 114 |
| New Lying-in Hospital, Dr. Beatty | 1 | ...... | 99 |
| Wellesley Dispensary, Dr. Maunsell | 1 | ...... | 34 |
| London West. Gen. Dispensary, Dr. Granville | 1 | ...... | 80 |
| Dr. Merrimann, (private practice) | 1 | ..... | 98 |
| Guy's Hospital Lying-in Charity | 1 | ...... | 101 |

In 3 cases only the long forceps were employed: in the remaining 6, delivery was effected by the short forceps. 5 children, out of the 9 delivered by the forceps, were still-born; and but 1 out of the 12 delivered by the vectis. The causes of perforation, in the 25 cases in which this operation was had recourse to, were, deformity, and abbreviation of the pelvic diameters; abnormally-developed fœtal head; an unusual degree of ossification; hydrocephalic head; rupture of vessel in the vulva and great effusion of blood, with subsequent laceration of mucous membrane.

A rather amusing and instructive case is detailed of a hydrocephalic head requiring puncture to admit of delivery. The mother had joined the tea-totallers during her pregnancy, and after confinement she gave them up as all moonshine—no one being able to pursuade her that the water in her child's head was not due to the immense quantity she had drunk!

In no case of protracted labour has there been long sloughing of the urethra, nor any case of fistulous communication between the urethra and vagina, or vagina and rectum.

*Preternatural Labour.*—The cases of preternatural labour amount to 109, or 2·33 per 100: the number of still-born children amount to 60, or 54 per 100. In Dr. Collins's report of the Dublin Lying-in Hospital, the number of preternatural labours amount to 506, or 3 per 100; the number of still-born children to 228, or 45 per 100. The nates-presentations were 59; and of these deliveries, 30 children were still-born. In the Dublin Lying-in Hospital, the nates-presentations amount to 242; the still-births to 73. In the Guy's Lying-in Charity, the footling cases were in number 29: the still-births 16. In the Dublin Hospital, the footling cases were 127, the still-births, 62. In the Guy's Charity, the presentations of the upper extremity were 15, the still-births, 10. In the Dublin Hospital the presentations of the upper extremity were 40; the still-births, 20. The funis-presentations amounted in Guy's Charity to 6: in 4, the children were still-born: whereas in the Dublin Hospital the presentations of the umbilical cord amounted to 97; the still-births to 73.

*Complex Labour.*—The number of cases of complex labour amount to 94; or 2·01 per 100. The number of still-births amount to 19; about 1 in 5, or 20 per 100. Of these 94 cases, 33 were twin labours; or 1 in 141 of the whole number of women delivered, being 0·7 per 100. The number of twins still-born amount to 6.

The following tables will shew the proportion of the sexes in twin labours, and also the nature of the presentations:—

| No. of Cases. | Both Males. | Both Females. | One of Each Sex. |
|---|---|---|---|
| 33 | 11 | 11 | 11 |

*Nature of Presentations.*

| | | | |
|---|---|---|---|
| Vertex in both | 15 | Vertex in both. Face in one situated externally | 1 |
| Vertex and Nates | 7 | Nates in both | 2 |
| Vertex and Foot | 5 | Nates and Foot | 1 |
| Vertex and Shoulder | 2 | | |

The proportion of twin-births is small, when compared with the returns of other Lying-in Institutions. In France, the proportion is said to be 1 in every 95 births; in Germany, 1 in 80; in England, 1 in 92; in Scotland, 1 in 95; and in Ireland, 1 in 62.

In 14 labours, continues Mr. Lever, presentation of the placenta has occurred: in 9 the placenta has entirely covered the os uteri, while in 5 it partially presented: in all, delivery was accomplished by the operation of turning. Eight of the children were still-born: in one, the head was unavoidably lessened, owing to the contracted state of the pelvis: (this operation had been resorted to in her previous labour). Presentations of the placenta have been more than four times as numerous in the patients of the Lying-in Charity of Guy's as among those of the Lying-in Hospital of Dublin: for whereas, at Guy's, 14 cases have occurred among 4666 women, only 11 cases in 16,414 occurred at the Dublin Lying-in Hospital during Dr. Collins's Mastership; and out of the eleven, 5 children were still born, 2 of them being putrid.

*Retained Placenta.*—These cases, amounting to 37, include those where the placenta is not expelled from uterine inaction, as well as those where there was morbid adhesion. 15 cases of the latter are recorded; and it is worthy of notice, that in 12 out of the 15, the placenta was adherent to the anterior and upper part of the uterus, as ascertained by the introduction of the hand to effect the removal. In the former cases, the uterus was stimulated to contract, in some instances by the introduction of the hand, but in the majority by the exhibition of the secale cornutum.

*Puerperal Convulsions.*—Four cases of this distressing complication have occurred; being in the proportion of 1 to 1166. The particulars of the four cases are briefly recited. In all, the children were born alive, and the mothers recovered.

Dr. John Clarke, in his abstract of 10,387 cases, has 19 cases of convulsions, 17 before delivery, and 16 cases of first pregnancy: 6 of the mothers died. Dr. Merriman states, in his "Synopsis, p. 141," that 28, out of 36 cases of convulsions which he had witnessed, were instances of a first pregnancy; and that in two of that number there were twins. Dr. Collins states, that 30 cases occurred, during his Mastership, among 16,414 women: 29 were in women with their first children; and the remaining case was a second pregnancy, in a woman who had suffered in a similar manner in her first labour. The 30 women attacked gave birth to 32 children, two of them having had twins. 14 children were born alive: 20 of the children were males: in 18 cases of the 30, the convulsions subsided after delivery: in 10, the fits occurred both before and after; and in 2, the attack did not come on till after delivery. Five of the women died.

Of *Epileptic Mania,* always occurring after delivery, there was a fatal case, and there was a case of *Puerperal Mania,* which was sent to Bethlem.

*Labour complicated with Typhus Fever.*—Of this there was an interesting case which did well.

*Rupture of Uterus.*—Three cases of ruptured uterus are reported as having occurred during the seven years embraced in this Report: they hold a proportion to the other cases, as one in 1555 cases: they all occurred in one year, 1839-40; and two within three days of each other. All proved fatal. They are related circumstantially, but we need not introduce them. Mr. Lever remarks on them.—

"These cases are replete with interest. In the first, the only one examined, there was old chronic disease of the womb; and at that part where the uterine tissue had degenerated, the laceration had occurred. In the second, every thing that could possibly be administered was done for the patient, with the exception of the employment of the ant. pot. tart. Should such a case again present itself, I shall most certainly prescribe the tartar emetic, in full nauseating doses. This remedy, of all others, seems to have the greatest power in controlling or cowing uterine action; and since the occurrence of this case, I never allow myself to be without some of the mineral in my possession. In the third case, it is hard to say at what time the laceration took place; as none of the symptoms occurred suddenly, but came on very gradually, and almost imperceptibly; so that it was probably only owing to my going fresh and unbiassed to the case that I was led to suspect the occurrence of an accident which Mr. Wright, an able and cautious pupil, had not imagined. For my own part, I cannot but believe that the violence to which this poor woman was subjected by her Irish charmer was the immediate and sole cause of the laceration."

The *charm*, to which Mr. Lever alludes, consisted in the gentleman's getting upon the bed, raising the woman to an upright position on the floor, and then lifting her up and down four or five times like a paviour's rammer, uterine efforts continuing all the while!

*Flooding Labour.*—The number of cases of flooding labour amounts to 51, or 1.09 per 100. Under this division are included 16 cases of accidental hæmorrhage before the birth of the child; 20 cases of hæmorrhage after the birth of the child, and before the expulsion of the placenta; and 15 cases occurring after the expulsion of the placenta. In the 16 cases of accidental hæmorrhage, rupture of the membranes was successfully practised, and only 2 children were still-born. In 12 of the 20 cases of hæmorrhage before the expulsion of the placenta, manual assistance was had recourse to, assisted by the exhibition of the different forms of the ergot already mentioned, and the other adjuvatory measures so well known.

*Fatal Cases.*—The number of fatal cases that have transpired during the seven years, is 40, or about 1 in 117. At the Dublin Lying-in Hospital, the proportion of women dying in child-bed was 1 in 100. A good and useful abstract of the cases is furnished, but we refer our readers for it to the original. This statistical paper reflects credit upon Mr. Lever.

### IV.—Report on the Value of Electricity, as a Remedial Agent in the Treatment of Diseases. By Golding Bird, M.D., A.M., F.L.S., &c.

Our able friend Dr. Bird has conducted a series of experiments on the remedial effects of electricity with great care, and his report is likely to be very serviceable. "In October, 1836," he says "the Treasurer, Mr. Harrison, appropriated an apartment in the hospital for the purpose of submitting patients to electrical treatment. In this room, all the apparatus required for the administration of electricity to the sick is kept in a condition fit for immediate use. The out-patients, as well as those from the various wards, attend at this room daily, at three o'clock; notes of their cases are taken by one of the Students, who acts as clinical clerk, and, in my absence, directs their treatment. A competent female attendant resides in apartments attached to the room, for the purpose of keeping the apparatus in order, and applying electricity to the female patients. In this manner, every case is registered; its progress watched by the pupils; and any change of treatment is directed by myself, for which purpose it is my duty to attend on two days during the week."

After pointing out the identity of electricity and galvanism, and stating the distinction which exists between them—viz., that whilst in both we have the very same form of matter, in the former we have a small quantity very concentrated, in the latter a much larger proportion in a dilate form. Dr. Bird goes on to remark :—

"When a person is placed on a stool with glass or other non-conducting legs, and connected with the prime conductor of an electrifying machine in action, his whole surface becomes, by induction, powerfully electro-positive; and combination, by silent discharge, is constantly occurring, by absorption of negative electricity from the air: this may be seen to take place luminously in the dark, especially about the hair, eyelashes, fingers, &c. During this luminous discharge, heat is evolved, the circulation becomes quickened, the secretions generally become more active, and perspiration breaks out. A person thus situated is said to be in an *electric bath;* and it is by no means improbable that this might be frequently employed with advantage in certain affections in which the functions of the skin and mucous membranes are deficient. The evolution of heat during this silent discharge is sufficient to demonstrate the mode in which the physiological effects alluded to are produced, and was, I believe, first pointed out by a former pupil of my class, Mr. Thomas Smith, now of Maryport, who performed the duties of clinical clerk in the electrical room, with the greatest zeal and attention, during several months. This gentleman coated the bulb of a delicate air-thermometer with tinfoil, and connected it with the conductor of an electrifying machine: silent discharge took place from the coated portion; and the coloured fluid fell in the tube upwards of an inch, from the expansion of the included air by the heat evolved.

If, whilst a person is connected with the prime conductor, and his whole surface is highly positive, or exposed to the *electric bath,* any conducting body connected with the earth, as the hand or a metallic ball, be brought near him, it becomes highly negative by induction; and when sufficiently near, the negative electricity combines with the positive of the person's body, producing a vivid flash of light and a snapping noise, forming the disruptive discharge, or what is called *the electric spark.* The person who is the subject of this experiment feels a sharp pungent sensation at the spot where the spark occurred; and in delicate skins, a small circumscribed wheal is produced, surrounded by a little inflammatory blush: this much resembles lichen urticatus, and generally disappears within an hour from the time of its production.

If an electric jar be charged, and the communication between its outer and inner surfaces be made through the medium of the human body, a peculiar stunning sensation, known as the *electric shock,* is produced. This seems to depend upon the neutralization of the electric condition of the jar producing disruptive discharge; and the physical conditions for its production are identical with those required for the spark, from which it differs only in its more intense effects, arising from the larger surface under inductive influence.

When the influence of a large quantity of electricity is deemed desirable, we have recourse to that form denominated galvanic or voltaïc electricity. This is readily excited by various forms of chemical action; but, for medical purposes, is more conveniently procured in a state of high tension by means of magnetic or electro-dynamic induction, various contrivances for effecting which are described in all works on Physics. The apparatus I prefer is that described in a Paper I published some few years ago; and may be briefly described thus :— A bundle of soft iron-wire is placed in the centre of a wooden reel or bobbin; around which is wound a quantity of stout insulated copper-wire, having its two ends free, for the purpose of being connected with a single pair of plates of copper and zinc exposed to chemical action: over this is wound about 1300 feet of very thin insulated copper-wire. The two ends of this coil are furnished with directors, for the purpose of being applied to any portion of the body or limbs required. If, then, one end of the inner coil be removed from the battery, the constraining inductive force previously exerted on the thin coil is removed, and all the electricity naturally present in it is discharged through the portion of the body or limb touched by the director, producing an electric shock. By adapting a piece of apparatus for mechanically breaking contact with the simple battery employed, a series of shocks can be sent through a limb, either very slowly and mildly, or at the rate of two thousand or more in a minute; producing such a rapid succession of discharges, as to give rise to a sensation amounting to insufferable torture.

In one or other of the three latter modes, electricity has been employed at the hospital; viz. in sparks, shocks from a jar, or shocks from the electro-magnetic apparatus last referred to.

### CHOREA.

A considerable number of cases of chorea, or St. Vitus's Dance, as it is popularly termed, varying in intensity and in the circumstances connected with its cause and development, have been submitted to electrical treatment since the publication of Dr. Addison's Paper on the Use of Electricity in certain Spasmodic Affections, in a former number of these Reports. Of these cases, notes of thirty-six have been preserved in the Report Book; the histories of the others not being complete, from the irregular attendance of the patients. Dr. Bird presents a table of these cases. Of these 29 were cured—5 were relieved—one patient left from alarm—and one found no relief: Dr. Bird observes that it must be born in mind, that, in many of these cases, remedies were administered during the application of electricity; and thus the reports of such lose a certain amount of their value: but in the majority of instances, the medicines administered contemporaneously with the application of electricity were confined to occasional mild purgatives. It is indeed notorious, that some medicines of this class often produce a considerable alleviation in the paroxysms of chorea, and occasionally are sufficient to cure the disease without the aid of other remedies; but, still, every practitioner must admit that this is not the constant result: and it must not be forgotten, that, in very many of the cases here related, every variety of treatment had been tried before the patients were admitted into the electrical room of the hospital. Dr. Bird adds:—

"In every case admitted into the electrical room, I have confined the treatment to sparks taken in the course of the spinal column every alternate day, for about five minutes each time, or until the papular eruption makes its appearance. I have very frequently found the disease to be somewhat increased on the first employment of the electricity, from the alarm of the patient; but this rapidly subsides; and in general, merely with the occasional administration of a purgative, the cases have rapidly progressed towards cure."

Dr. Bird relates nine cases, and appends to them the following conclusions:—

"It may now be asked, in what light is electricity to be regarded, in the treatment of chorea, and certain involuntary motions of the voluntary muscles analogous to those occurring in this disease? From the results of the cases treated at Guy's Hospital, no doubt can remain, on the mind of any one, that electricity really exerts a decided, not to say specific, influence on these affections: and although, on its first application, all the symptoms often become increased, from, probably, the timidity of the patient, and the novel character of the remedy, yet, where it has been persevered in, in thirty-five of the thirty-six reported cases, it has either completely cured, or greatly relieved the patient: the case in which it failed, the 29th in the table, could scarcely be regarded as a fair one, as there was but little doubt that disease of the membranes of the spinal cord existed. As I feel extremely unwilling to recognise the existence of more specific agents than necessary, I would venture to suggest that electric sparks, when drawn from the spine, may act by the irritation they produce: and this appears countenanced by the fact, that the rapidity with which the patient's symptoms are relieved is nearly in a ratio with the facility with which the peculiar papular eruption makes its appearance. If future experience should countenance this view, the remarkable influences exerted by electricity in the treatment of chorea may be referred to the principle of counter-irritation. It may next be inquired, whether, in the treatment of chorea, we ought to trust to electricity alone? In answer to this, it may be stated, that I have repeatedly treated severe cases of this disease successfully with this agent, without the administration of any medicine at all; and in the majority of the thirty-six cases in the table, no internal medicine, except an occasional purgative, was administered during the electrical treatment. Still, it is obvious that, in many cases, the disease is kept up and excited by the irritation produced by some deranged function; and this of course it will become the duty of the physician to set right, before or during the application of the electricity. The case of Eliza Raven affords an interesting illustration of this: here the chorea either depended, *ab initio*, upon the non-performance of the uterine functions, or was kept up by it; and, accordingly, a few electric shocks through the pelvis, by restoring the deficient menstrual discharge, at once cured the patient."

### PARALYSIS.

Paralytic affections constitute a prominent feature in the cases which have been referred to the electrical room of the hospital; of these, forty-four cases have been fully reported by the clinical clerks, and may be found on record in the Hospital Books. Of these cases it may be generally remarked, that those in which the paralysis, whether of sensation or motion, or both, depended upon exposure to cold or rheumatism, upon some functional affection often of a local character, or upon the impression produced by effusion in some part of the cerebro-spinal centre, which had become absorbed under the influence of previous treatment, the result of the application of electricity was most successful; whilst in those cases in which the paralysis depended upon some persistent structural lesion, whether produced by accident or otherwise, Dr. Bird has never seen the slightest benefit result.

The reported cases of paralysis may be divided into those of dropped hands, those of a rheumatic character, those depending upon some lesions of the cerebro-spinal centre, upon local injury applied to a limb, and upon hysteria. Of dropped hands there were eleven cases. Five were cured—three were relieved—one improved—and two experienced no relief.

In the treatment of these cases, electricity was generally employed in the form of sparks drawn from the upper part of the spine, so as to exert its influence over the origin of the spinal nerves forming the axillary plexus. In the majority of cases, medical treatment was also employed; as, in general, the subjects of this particular disease are always deranged in health; the functions of the digestive organs being imperfectly performed, especially as in most the patients had been previously the subjects of colica pictonum.

In cases where the general health was not much deranged, the use of electricity over the spine, and drawing a few sparks occasionally from the paralyzed extensor muscles of the wrist and hand, with the exhibition of an occasional laxative, was generally remarkably successful. But in those instances in which the patient was of cachectic habit, and the constitution deranged by previous or existing functional derangement of the digestive organs in particular, electricity was of no service, or, at most, of doubtful efficacy, until the deranged functions had been relieved by the remedial measures employed.

Dr. Bird relates four cases of paralysis of the extensors of the hand. An observation in connexion with such a case in a compositor deserves notice. The types are occasionally wetted, for the better distribution into their respective boxes. And if the damp types are imprudently exposed to the fire, for the purpose of drying, an empyreumatic acid matter appears on the surface of the types, and is probably absorbed by the compositor's fingers. If this very reprehensible and unnecessary drying is avoided, no injury arises from the use of types.

"In cases of the dropped hands of painters, the conditions before mentioned being borne in mind, the electric sparks drawn from the region of the cervical and dorsal vertebræ are generally efficacious in at least aiding, if not effecting, a recovery. I have generally, also, directed them to be drawn from the paralyzed parts; and, in recent cases, small shocks, transmitted along the course of the affected nerves, have considerably accelerated convalescence: but in chronic cases I have repeatedly seen a cure effected by drawing sparks from the spine, on alternate days, for weeks, after shocks had been passed along the paralyzed parts in vain."

*Rheumatic Paralysis.*—Cases of this affection, says Dr. Bird, are by no means unfrequent in practice, and are readily distinguished, on careful investigation, from those which depend upon cerebro-spinal lesion. The cases most readily confounded with them are those in which the legs or arms appear paralyzed, but in which the inability to move the limbs depends rather upon the pain produced by motion than upon any real want of power. The common history of these cases is, that a person after exposure to damp and cold, and sudden alterations of temperature, suffers from a slight febrile attack, followed by inability to move one or other of the limbs, and often a single leg or arm, if either of these have been exposed to the influence of a draught or current of air. In general, sensation remains either slightly or not at all impaired, but the paralysis of motion is generally tolerably perfect. This state may continue for an almost indefinite period; and at length, from want of exercise, the muscles of the affected limb become atrophied, and the chance of relief from treatment of any kind becomes proportionably diminished. In cases of this kind, before this wasting has occurred, the influence of electricity is very remarkable, frequently restoring power to the paralyzed muscles in a very short time.

We have a table of ten cases, and memoranda of four.

*Paralysis from Affection of the Nervous Centres.*—A considerable number of patients, labouring under various forms of paralysis, depending upon some functional or structural lesion of the great nervous centres, have been admitted under treatment at the electrical room. Of these, many were of that character in which no rational hope of relief could for a moment be entertained: of this class were those in which the paralysis depended upon some persistent lesion, as the pressure of an effused clot, of a tumor, or of bone on the surface of the brain or spinal marrow. And many of these cases, in which electricity afforded no relief, were subsequently submitted to post-mortem examination; and in all, the existence of some persistent cause of paralysis was distinctly made out. Dr. Bird has made out a table of 13 cases in which relief might fairly be expected. Of these, six were cured, two relieved, and five were not relieved. Seven of the cases are detailed. We quote one case—a striking one.

*Case.*—Stephen Burn, aged 11, admitted Jan. 15, 1840, into the hospital, under Dr. Addison, labouring under total loss of motion of the right leg and side, which appeared seven weeks previously, without any very apparent cause, whilst in bed. He has been gradually getting worse; and has been cupped, taken mercury, his head shaved, &c., without any marked benefit. He was carried from the ward into the electrical room, being quite unable to walk.

Sparks were freely drawn from the spine and affected limbs. The effect was remarkable; for the boy almost immediately recovered power over the previously paralyzed side, and he walked back into the ward with only the aid of a stick. After attending a few days longer, he was presented completely well.

*Paralysis from Local Injury.*—Several cases of paralysis of a single limb, or part of one, from local injury, have been submitted to the action of electricity. Four cases are alluded to—one, paralysis of the left arm from falling on it—the second, paralysis of the right arm from injury to the shoulder—the third, paralysis of the ankle joint from falling with the foot bent—the fourth, paralysis of the left arm from dislocated humerus. Of these cases, says Dr. Bird, it may be briefly observed, that in those in which there was evidence of positive structural lesion of one or other of the nervous trunks supplying the affected limb, no benefit resulted from the application of electricity; whilst, on the other hand, where the nerve had been left only functionally paralyzed, and its structure unaffected, an electric current produced rapid, and in some instances immediate, relief. Of the four cases inserted in the above Table, the third and fourth were instances in which there could be but little doubt that the nervous trunks were themselves mechanically injured: in the fourth case in particular, in which a man dislocated his humerus into the axilla from a fall, and it remained unreduced for seven weeks. On his admission into the hospital, under the care of Mr. Cock, the dislocation was reduced, but the arm was left in a partially-paralyzed state; the parts supplied by the internal cutaneous and median nerves being especially affected. In this case, as might be expected, electricity was found altogether useless, as there was reason to believe that some branches of the axillary plexus had become structurally injured by the pressure to which they had been subjected.

Two cases are related more in detail, but we need not introduce them. Dr. Bird goes on to remark—"In these curious cases of partial paralysis, in which we find the affection limited to a very small portion of a limb, often to a single muscle, I have found the application of electricity of considerable service; although, from the rarity of these cases, I have not had sufficient experience to allow me to offer any thing more than a very general opinion. In the few instances of this local paralysis that have fallen under my notice, the power of motion has been alone lost, sensation having continued tolerably perfect; and the local application of the current from the electro-magnetic machine has been generally successful: in addition to this local treatment, the general health of the patient has been attended to, by the administration of appropriate medicines, and a vigilant attention to diet."

The following case is not uninstructive:—

CASE.—*Paralysis of Motion, limited to the Biceps Flexor Cubiti.*—Dr. Bird was consulted in March, 1839, by a gentleman of about 40 years of age, of sedentary habits, for a loss of power in the left arm: he had considerable difficulty in flexing the limb, and was quite unable to raise the fore-arm to any considerable extent. On examining the limb, the belly of the biceps was found remarkably atrophied, being scarcely one third as much developed as in the corresponding muscle of the right arm; the brachialis anticus appeared not to participate in this change, at least to any considerable extent. Sensation appeared to be tolerably perfect in the affected muscle. The patient stated, that this loss of power had been insidiously coming on during some months, and could offer no probable explanation of its origin. Dr. B. fancied it might be attributable to exposure of the arm to a draught of air. The general health seemed good. Blistering over the biceps and strychnia were of no service, and electricity was resorted to. The patient provided himself with the electro-magnetic apparatus, and passed a current from the region of the cervical vertebræ to the belly of the muscle, daily:

this always produced violent contractions and spasmodic movements of the arm. By continuing this treatment during several weeks, power gradually returned to the muscle, and in the course of some months, he had as much power over the flexors of the fore-arm as ever.

"The cases here recorded are, I think, sufficient to justify the conclusions I have drawn of the value of electricity as a remedial agent in paralysis; and I have no hesitation in stating my conviction, that, in the majority of cases, a careful investigation into the history of the ailment will enable the practitioner to determine whether electricity will be of service in any particular case. Every one must be aware of the occurrence of cases in which paralysis generally affecting one side of the body, comes on as a mere effect of congestion or of effusion, which, under the action of depletion and mercury, can be completely removed; and yet the effect of such morbid action, the paralysis itself, remains. Cases of this kind will generally do well by time, aided by the application of friction, baths, exercise, &c.; yet convalescence will be extremely protracted. Here the passage of a few electric shocks through the limbs, so as to arouse, as it were, the dormant functions of the nerves, will at once frequently restore as complete power over the affected limb as the state of the muscles, weakened by previous disease, will admit of. Of this we have a remarkable instance in Case 22. When the paralysis has been of longer date, or the cause producing it of a more persistent character, it will be found necessary to continue the application of the electric shocks for a longer period of time. In these cases, it will generally be found more convenient to allow a rapid succession of shocks to pass through the paralyzed limbs, by means of the interrupted current from the electro-magnetic machine. Indeed, so far as my experience has extended, I am far more inclined to place reliance on the current of electricity yielded by this machine, than by that obtained by means of friction; as we have at our command an enormous quantity of electricity, of high tension: added to which, its application is much more convenient, and requires less tact than when the common electric machine is employed. The truth of the statement here made, of the class of cases of paralysis in which electricity may be employed with every probability of success, is supported by what we see in its employment in the local paralysis following the application of mechanical injury to a limb: for wherever the nervous trunks supplying a limb are lacerated, or otherwise mechanically injured, we can hold out no hope of relief from the application of electricity, or any other remedy; whilst in those (as Cases 25 and 26) in which, from the shock of the blow applied to the limb, the nervous trunks had been only functionally paralyzed, their structure remaining unaffected, the passage of the electric current affected a complete restoration of the previously deficient nervous power of the limb.

In accordance with this reasoning, we find those anomalous forms of paralysis connected with Hysteria generally yield to the application of a few electric shocks. I have, indeed, repeatedly seen hysteric paraplegia yield so rapidly to the effect of an electric shock, as strongly to impress those who were watching the case with a conviction that the whole disease was simulated."

And we have no doubt that simulation, or something not a hundred miles removed from it, has a great deal to do with many of these cases.

### AMENORRHŒA.

"Scarcely any cases have been submitted to electrical treatment in which its sanatory influence has been so strongly marked as in those in which the menstrual function was deficient. The remarks previously made regarding the electrical treatment of chorea and paralysis alike apply to amenorrhœa: so long as the patient is seriously out of health, as when marked symptoms of chlorosis are present, scarcely the slightest benefit has ever resulted from the employment of electricity: in fact, as this agent can in these cases be regarded but as a local stimulant applied to an organ whose function is deficient, we could hardly expect the menstrual discharge to appear, when, from the deranged state of the general health, the womb is not in a state to supply the deficient secretion. The rule for insuring success in the great mass of cases of amenorrhœa is sufficiently simple:—improve the general health by exercise and tonics; remove the accumulations often present in the bowels by appropriate purgatives; and then a few electric shocks, often a single one, will be sufficient to produce menstruation, and at once to restore the previously deficient function. It is for want of attending to this rule that so many cases have been said to have been unsuccessfully treated by electricity; and to this statement I must oppose all the experience acquired from the cases treated in the electrical-room of Guy's Hospital; for, with but one or two exceptions, every case in which the general health was not too severely deranged, as by chlorosis, has been successful; of course, not including those who, from timidity or other causes, never appeared but once or twice at the hospital."

Dr. Bird recommends the following method of applying the electric shocks. Let the patient be placed on a chair or stool; press the brass knob of a director against the sacrum; and if the stays be loosened, to that only the linen intervenes between the latter and the knob, no further exposure is necessary. A second director, furnished with a chain connected with the outside of an electric jar, is passed by the female attendant under the patient's dress, and the knob pressed against the pubes. The jar is then charged; and its ball touched by a third director, connected with the one held against the sacrum by means of a chain. The shock thus passes through the patient's pelvis; and should be repeated ten or a dozen times. The jar employed should hold about a quart, and be about half charged.

A Table is given of 24 cases of amenorrhœa. Twenty were cured—four were not relieved. The latter patients were the subjects of well marked chlorosis, and of an irritable condition of the uterus. " In general," says Dr. B. " whenever the menstrual discharge has appeared under influence of the electric treatment, I have directed the remedy to be intermitted as soon as the flow has been fairly established, and its use to be recommended about a week before its expected return, as in Cases 33, 34. Whenever, as has occasionally occurred, the use of electricity has been persisted in during four or five weeks without benefit, I generally advise discontinuing it altogether; and by a careful investigation of the case, to ascertain, as far as is possible, the cause of the deranged general health, which always, so far as I have seen, exists in these unsuccessful cases: and after removing this by remedies directed to the particular functions involved, the use of electricity will then seldom fail to restore the catamenia, and thus effect the cure of the patient."

### OTHER DISEASES.

The results, in these, have not been sufficiently uniform to warrant any trustworthy induction to be drawn. In amaurosis, especially, the results of the trials were too conflicting to afford any satisfactory conclusions: in the majority of instances, it certainly did no good: in a very few, it appeared to afford some relief; but in none did it effect any thing like a cure. In various cases of deafness the results of the application of electricity were similar. It is of course more than probable, that as amaurosis and deafness are in themselves but symptoms, various totally distinct affections of the eye and ear were submitted to treatment, and hence could afford no satisfactory conclusion.

In chronic rheumatism, especially in sciatica and lumbago, a considerable alleviation of suffering was frequently effected by drawing sparks from the seat of pain; but the cases submitted to treatment have not been as yet sufficiently numerous to warrant any general conclusions. Dr. Bird has never seen it of the slightest benefit in ovarian dropsy.

Nor has he observed it to be serviceable in epilepsy—nor, indeed, in the treatment of convulsive affections in which the brain was involved, as in epilepsy; whilst, as already stated, in chorea, in which the brain is generally unaffected, the result of the application of electricity was most gratifying.

In hysteric epilepsy occurring in women in whom the uterine functions were suspended, electricity, by restoring the catamenia, was of material service: these can, however, be scarcely regarded as cases of genuine epilepsy, but rather of an hysteric convulsion, depending upon the non-performance of the uterine functions. Several cases of this description have occurred at the hospital. The following is an instance.

Case.—Sarah Watson, aged 15, a stout girl, of remarkably dull appearance, states, that six months before admission into the hospital she was in possession of tolerably good health. At that time, however, she became the subject of fits of an epileptic character: these occurred at irregular intervals, and lasted each time about fifteen or twenty minutes. This girl has never menstruated; and she labours under the nervous feelings and headache, so frequent in amenorrhœa.

*Oct.* 10, 1839. Shocks through the pelvis, thrice a-week.

13. The shocks were passed this day for the second time, and in a short time were followed by pains referred to the back and loins: soon afterwards, the menstrual discharge appeared.

14. Catamenia continue flowing.

From this time she remained free from fits.

A very useful paper.

### V. Observations on Real and Supposed Pathological Conditions of the Urine. By G. O. Rees, M.D.

In the Medical Gazette, Dr. Rees recently noticed a peculiarity in the urine passed by patients taking copaiba; such urine becoming coagulated on the addition of nitric acid, notwithstanding that no albumen was present. He has since found a very similar condition in the urine of persons taking cubebs.

The urine impregnated with copaiba always yielded a precipitate, on the addition of nitric acid: in some cases, this was very slight; but in the majority, very dense and white, and greatly resembling albumen. The action of the acid on urine impregnated with cubebs is precisely similar in character, excepting that the colour of the precipitate occasionally verges on a pale pink. Previous to his publishing the re-actions of copaiba, there was but one substance (albumen excepted) supposed to be precipitable from the urine by nitric acid: this was lithic acid, which, however, could not be regarded as a source of fallacy; as, when thus precipitated, it comes down only after the tested liquid has been allowed to stand some time, and then appears in a semi-crystalline form, and of a brownish-red colour. The precipitate afforded by cubebs and copaiba is, however, of that cloudy opaque character which simulates albumen, and moreover occurs immediately on adding the test.

It became an object now, to discover some simple method of discriminating between this precipitate and that obtained by nitric acid from albuminous urine. Fortunately, a very easy means of doing so is afforded us, by allowing the urine to which the acid has been added to remain at rest for an hour or two; when, should the precipitate consist of albumen, it will be found to have collected at the bottom of the tube, or to be arranged in flocculi through the liquor, the greater part of which will appear clear. If, however, the precipitate be caused by the presence of a vegetable matter derived from copaiba or cubebs, the precipitate does not subside for several days; not, indeed, till decomposition has occurred. Another and more speedy method of discriminating between these two impregnations and albumen is by the use of the ferro-cyanuret of potassium as a precipitant, the urine being previously acidulated by acetic acid. If albumen now be present, it is immediately thrown down; but in the other cases, even if the acetic acid cause a slight turbidity, it is not increased by the addition of the ferro-cyanuret. It is interesting to observe how supposed exceptions to the law declared by Dr. Bright become explained away by advances in the chemical pathology of the urine; copaiba, for instance, has been stated to cause albuminous urine; and probably cubebs may also have been looked upon as capable of affording the symptom which Dr. Bright has regarded as characteristic of a peculiar morbid tendency in the kidney.

Dr. Rees dwells on the necessity for using both nitric acid and heat as a test for albumen. Yet the former may thus throw down a precipitate from vegetable matters not albuminous, and the latter may throw down the phosphates.

"I must mention, that the conditions I have noticed are not always to be expected, either in the case of copaiba or cubebs; appearing most strongly when the urine smells powerfully of the drug, and being scarcely perceptible when the characteristic balsamic odour is wanting. I have reason to believe, that when either cubebs or copaiba are administered with an alkali, that the urine becomes more rapidly and completely impregnated: but it is difficult to conceive what cause can be in operation to produce an occasional difference in this respect, the urine suddenly becoming (even when large doses of the medicine are being exhibited) comparatively free from impregnation."

Dr. Rees goes on to remark :—

"The frequent occurrence of the earthy phosphatic precipitate afforded by the action of heat on urine, and, consequently, the fallacious nature of the test by heat, may be better appreciated, if I now lay before the reader a statement drawn up from some valuable Tables which were formed by Dr. Barlow and Mr. Tweedie, at the suggestion of Dr. Bright. I find, by examining these Tables—in forming which, the tests both of heat and nitric acid were used to ascertain the presence of albumen—that in 482 cases, taken promiscuously from the hospital wards, 34, or about 7 per cent, were found which coagulated or became opalescent by heat, while they were not affected by nitric acid; these, therefore, were cases in which the phosphates were precipitated; and had heat alone been used as a test for albumen, we now observe how many errors must have been committed, and how many cases might have been cited as exceptions to Dr. Bright's law. Though well aware of the frequent occurrence of this source of fallacy, still I must say, I was scarcely prepared to find it in so large a proportion of cases as 7 per cent. In Tables constructed by Dr. Barlow, in which he examined the urine of 300 individuals, we find that 1 in 11 had albuminous urine: other tables make 1 in 6 as the proportion; the former being about 9, and the latter nearly 17 per cent. This shews how impossible it is to arrive at a satisfactory approximation to the truth by the use of the test by boiling alone: for among 100 persons, 16 might be declared to have albuminous urine; and of these, only 9 might present the degeneration of kidney on post-mortem examination, the 7 cases opposing the general truth which the observer was seeking to confirm."

Dr. Rees thinks it may be concluded that the phosphates are apt to be precipitated from urine, on the application of heat, in patients of a cachectic habit, and in those worn by disease and suffering. The specific gravity of such urine is often as low as 1010 and 1012: though this is not always the case, as we sometimes find it natural, viz. from 1017 to 1022.

Dr. Rees has also examined into the assertion that patients in a state of salivation present albuminous urine. Mr. D. Francis has constructed a Table of 15 cases of salivation.

Dr. Rees adds:—

"From these observations we may safely conclude that mercury does not always produce albuminous urine: and though we cannot say, from these few instances, that it never is a cause of the existence of that principle in the excretion, yet these cases may serve to warn the reader from depending too much upon loose assertions. I have myself observed that, urine containing albumen sometimes becomes freed from that substance by exhibiting mercury to the patient. Mr. Francis, during his late inquiries, met with such a case: and I quote the following from a note I received from him.

'The other case was one of albuminous urine. The patient was salivated: and whilst under the mercurial influence, the albumen entirely disappeared.'"

### VI. Some Observations on Stricture of the Urethra, Catheterism, and False Passage: founded on Post-Mortem Inspections. By Edward Cock.

The object of Mr. Cock is to shew how much violence and how much injury is daily done to the urethra by the forcible introduction of instruments. Of this there cannot be a doubt. False passages are continually made—great irritation is set up in the whole line of the urinary canal—perinæal abscesses, or even serious extravasation of urine, are the consequences—and a life of suffering, or a premature death, are the effects of a want of knowledge, dexterity, or patience, in the surgeon.

Mr. Cock shews, by cases, that the canal is constantly damaged, even when the instrument is got into the bladder—how much more so when it is not. "The surgeon," he observes, "when applied to in a case of difficult micturition or complete retention, congratulates himself and his patient on having succeeded in passing a catheter through a canal which, perhaps for months previously, has only allowed the escape of a slender thread-like stream, or possibly a mere dribble: he is said to have expanded the stricture. Sometimes the introduction is accomplished at the expense of much bloodshed: he is then said to have broken down the stricture: by which, I suppose, is meant the tearing down one side of the wall forming the narrowed portion of the canal, and thus laying it open, as it were, into the track of the instrument. The main object, however, has been accomplished; the catheter has entered the bladder; and the satisfaction experienced by the patient and the operator may probably render the latter unwilling to investigate the exact manner in which his success has been brought about. Nevertheless, the moderate permanent good, and the frequent mischief, which result from the passage of instruments, even where we have succeeded in entering the bladder—the scalding pain and rigors which accompany and follow micturition—and the not unfrequent occurrence of perinæal abscess resulting from the passage of a catheter—must, upon reflection, lead us to the opinion that the instrument has overcome the difficulty in a manner different from that which we had contemplated."

Mr. Cock lays before us some cases which have occurred only during the last twelve months. They are not picked cases of false passage, but comprise all the instances in which death occurred, from different causes, shortly after the introduction of a catheter *into the bladder*, where he could make a post-mortem examination. These cases we shall abbreviate.

*Case* 1.—A. D. aged 60, died of suppuration round the hip-joint. About a fortnight before his death, an abscess formed in the perinæum, which was opened, and discharged a quantity of fetid pus mixed with urine. It was then ascertained that he had laboured under stricture for many years, voiding his water by dribblets. He now passed his urine freely through the wound in the perinæum; but in a few days' time, as he appeared to be again suffering from retention, a small catheter was introduced into the urethra, and passed without much apparent difficulty into the bladder, when a considerable quantity of water was drawn off. The silver catheter was replaced by an elastic one, which he wore until his death. On laying open the urethra, it was found contracted opposite the bulb, to the extent of about two lines. A small probe could not be passed through the stricture, even under the application of considerable pressure; but a punctum probe passed readily. Immediately behind the stricture was an ulcerated opening, communicating with the perinæal abscess. The catheter had penetrated the walls of the urethra just anterior to the stricture, and formed a false passage along the upper part of the canal, merely separated from it by the lining membrane, until it entered the bladder above the prostate.

*Case* 2.—W. B. died from the effects of retention, followed by extensive extravasation and sloughing, extending through the perinæum and scrotum, and over the abdomen. Some days before his death, a sound was passed into his bladder. A firm cartilaginous stricture, which would just allow the passage of a probe, existed in the membranous portion of the urethra, behind which the canal was dilated into a pouch. The sound had evidently passed through the walls of the urethra anteriorly to the obstruction; but in what manner it had entered the bladder could not be ascertained, in consequence of the diseased condition of the parts.

*Case* 3.—W. C., aged 53. The urethra opposite the bulb was found nearly obliterated by a stricture, surrounded by extensive induration: behind it the canal was perforated by an ulcerated opening, through which the urine had escaped, and produced the extravasation. The instruments which had been introduced had left the urethra anterior to the stricture, had apparently passed through the softened and broken-down structures, and had re-entered the canal close to the prostate.

*Case* 4.—G. L., aged 71. A moderate-sized catheter was passed, his urine was drawn off, and the instrument was allowed to remain in the bladder for three days, when it was withdrawn; and he subsequently got rid of his water in a small stream by natural efforts. At the post-mortem inspection, the urethra was found relaxed and flabby, but capacious as far as the membranous portion, where it suddenly became contracted for a couple of lines, so as just to admit the passage of a sound, No. 3. The catheter which had been introduced had not penetrated the stricture, but had passed by the side of it, and been carried between the canal and the pubes until it penetrated the upper part of the prostate, and entered the bladder just above the natural entrance to the urethra. The track of the instrument presented an ulcerating and sloughy surface, but no extravasation had taken place; and as, after the withdrawal of the catheter, he experienced no pain during micturition, it is probable that he continued to pass his water through the natural canal, and not through the false passage.

*Case* 5.—T. C., aged 67. The urethra was very capacious, except just beyond the commencement of the membranous portion, where a stricture existed, probably of long standing. The contraction was about two lines in extent, and just large enough to admit the smallest-sized catheter. There were two false passages, evidently of recent formation, through which his water had been drawn off since the first introduction of the catheter; for it was manifest that the strictured portion of the canal, which was surrounded by a slight induration, had never been entered, being far too small to allow the passage of the instruments which were used, and bearing no marks of having been dilated or passed through. The one false passage left the urethra about one inch and a half anterior to the stricture, to the left and upper part of the canal; passed just above the urethra for about three inches; and entered the upper wall of its prostatic portion. The other false passage left the urethra immediately anterior to the stricture, through a lacerated opening at the right and lower side of the canal; and re-entered the urethra close to the bladder, by piercing the prostate on the right side of the veru-montanum. A communication existed between these two false passages about the middle of their course, as if the catheter had been occasionally passed from one into the other. Thus there were four ways of entering the bladder, besides the right one.

*Case* 6.—"J. H., aged 60, died of ascites, for which he had several times been tapped. On the last occasion, previous to introducing the trochar, I attempted to pass a catheter; as he informed me he had long laboured under difficulty in voiding his urine. I found a stricture which I was unable to penetrate, and desisted from the attempt. On examination after death, I exposed a contraction of the urethra just opposite the bulb; and endeavoured to dilate it, first with a moderate-sized catheter, and afterwards successively with smaller ones, but found that the application of considerable force was insufficient to expand and penetrate the stricture. It allowed the passage of a large probe."

Mr. Cock remarks that this last case illustrates the physical difficulty, in many instances the impossibility, of overcoming an organic contraction of the canal by the mere application of temporary pressure. An old stricture cannot be taken by storm, and will not allow the passage of an instrument larger than the opening itself.

Mr. Cock offers a few suggestions.

The habitual size of the stream by which the patient voids his urine, and the effort and straining he is obliged to employ to get rid of it, are the best, and, in fact, the only means we possess, for arriving at an estimate of the dimensions of the strictured portion of the canal. In most instances, the longer the stricture has existed without proper remedial treatment, the firmer and more unyielding it will be found. This is the common sense view which we always act on, and which we believe to be the safest we can take.

Mr. Cock remarks that, in the ordinary directions for the introduction of a catheter, considerable stress is deservedly laid on the necessity for keeping the point of the instrument in the direct line of the canal: but we must not be too confident, that a strict observance of this rule will prevent us from occasionally eluding the stricture, and making a false passage into the bladder; as probably no appreciable difference will be recognised in the position of the instrument, whether it remains in the urethra, or is carried on just outside the membrane lining the canal. This we conceive is borne out by every practical surgeon's experience. However correct the position and destination of the instrument may seem, it may perforate the walls of the urethra notwithstanding.

After a catheter, continues Mr. C., has been successfully introduced into the bladder, it may often be a matter of some difficulty to determine, whether the object has been effected by carrying the instrument through the stricture, or opening a passage by the side of it. The condition of the patient subsequent to the introduction may assist us in arriving at an opinion. When, owing to the situation of the obstruction, a fortunate manipulation, or any other favourable circumstances, we have been enabled to introduce, without violence, without pain or loss of blood, a small catheter, which is suffered to remain for a longer or shorter period of time, we shall almost invariably find that the symptoms have undergone a material alleviation; that he passes his water in a larger stream with less difficulty and straining, and that the canal is in a condition to allow of a still further dilatation; in fact, that we have accomplished the first important step towards a progressively successful cure. On the other hand, our anticipations of a favourable change will generally be disappointed when the instrument has eluded the stricture instead of penetrating it. The introduction itself has probably been effected with some degree of violence, and is attended with pain and loss of blood. After the withdrawal of the instrument, the false passage may immediately close up, and the patient then remains in his previous condition; or the water may find its way in a reluctant stream through the new canal, accompanied by much smarting and irritation, and followed by rigors and constitutional disturbance: or a partial infiltration may, perhaps, take place, ending in the formation of a perinæal abscess. The catheter is introduced again and again, with similar results; until it is found advisable to suspend the mode of treatment, or, under more favourable circumstances, until the new line of road becomes sufficiently worn and polished by the instrument to take upon itself the functions of a urinary canal. We have no doubt that the rigors, &c. which have been regarded as almost natural consequences of the introduction of instruments, are, in many instances, at all events, the result of their too forcible employment.

"The opinion has been advanced and maintained, that a new canal may be established with impunity; that it becomes lined by mucous membrane, and for the future answers the purpose of a water-course to the individual, while the old channel becomes abandoned and filled up. I do not intend to deny that such a desirable consummation may occasionally take place; but the observations I have been enabled to make on the subject incline me to believe that the result of an 'error loci,' on the part of the surgeon, is more frequently disastrous than favourable to his patient.

The mischievous consequences which are likely to ensue after a false passage has been made into the bladder may depend on its situation, its extent, or the degree of injury which has been inflicted on the surrounding textures, and on the constitutional irritability of the patient. In some cases, as I have already stated, a short artificial canal may be effectually established by the side of the stricture, and the condition of the patient permanently benefited by this diversion of the natural course of the urethra. In other instances, the new passage remains open only so long as an instrument is retained in it; or it may continue pervious for a certain time, and allow the urine to flow through it until the vis medicatrix naturæ has gradually healed the wound inflicted by the surgeon; and then the old canal, including the stricture, is again brought into use;—the patient, of course, relapsing into his original condition, the difficulties of which induced him to seek advice and assistance. I believe that the train of events which I have just described not unfrequently occurs; and the unsatisfactory result is generally attributed to a tendency in the urethra to *resume* its originally contracted state; whereas, in all probability, the stricture, which is thus said to have become re-established, had never been at any time removed or dilated by the introduction of an instrument.

Some instances have come under my observation, of persons who had undergone a previous course of catheterism for the relief of retention and the cure of obstinate stricture; and who, as far as could be ascertained from the account which they gave, from a careful examination, and from the symptoms manifested, had apparently lost, by obliteration, a considerable portion of the urethra including the stricture; and acquired a new passage, in no way better adapted for the purposes of micturition. The walls of the canal were irregular and granular, furnishing a copious purulent secretion, and exquisitely tender and irritable. The urine was voided with difficulty and pain; violent spasms frequently induced; and the life of the individual rendered one of misery and apprehension, by repeated attacks of retention of longer or shorter duration. The disposition of the canal to close rendered the use of the catheter frequently necessary; and the introduction of the instrument, which could only be effected at the expense of great suffering, afforded but a temporary relief. Such a condition of parts hardly admits of any permanent alleviation, and gradually undermines the health of the patient; inducing disease of the bladder and kidneys, by which he is finally destroyed."

Mr. Cock concludes with two cases in point.

VII. Cursory Observations on some Cerebral Affections of Children. Read before the Physical Society of Guy's Hospital. By H. Marshall Hughes, M.D.

Dr. Hughes first adverts to "Infantile Remittent Fever," which, he thinks might be more correctly designated—"irritative fever of children." After a slight sketch of the symptoms and some judicious observations on the treatment, Dr. Hughes goes on to say,—but in the course of this affection—which, by a high authority, has been mentioned as "remarkable for being always devoid of danger," and under circumstances undistinguishable from those accompanying the ordinary form of the complaint—the physician is not very unfrequently informed that his patient has been suddenly seized during the night with violent pain of the head, screaming, and convulsions; and, in the morning, finds it labouring under sufficiently-marked symptoms of arachnitis; as severe pain of the forehead, heat of the scalp, intolerance of light, contracted pupils, vomiting, a bounding but perhaps not very frequent pulse, and other indications of cerebral excitement, which need not be enumerated. He is then led to doubt if he may not previously have mistaken the nature of the case; and whether, from its commencement, it may not have been one of slowly progressive meningitis or hydrocephalus. He thenceforth directs his principal attention to the brain: bleeds, leeches, and purges; applies blisters, and administers calomel assiduously; but too often without any permanent benefit. The disease, after many fallacious appearances of amendment, passes on to effusion into the ventricles, from which the patient dies; and, on examination after death, are discovered the ordinary consequences of inflammation of the brain or its membranes.

Dr. Hughes relates three cases which it is unnecessary to insert, and contrasts them with two other cases of pure inflammatory affection of the brain and its membranes. Dr. Hughes contrasts two descriptions by two authors—one of acute hydrocephalus—the other of infantile remittent fever; and although the liability of the diseases being confounded is not dwelt on by those authors, the descriptions of the two disorders are wonderfully alike. Dr. Hughes then observes—

"Certain questions then arise, to which I am anxious of directing the especial attention of the Society; and the correct decision of which is most important. Are the two or three cases which I first mentioned, and all cases of a similar character, examples of simple remittent fever, in the course of which arise symptoms of arachnitis; or are they, *ab initio*, cases of slowly progressive inflammation of the membranes or substance of the brain? If the latter is the correct view of their pathology, it may be asked, How are they to be distinguished from ordinary cases of remittent fever? Or if the former is regarded as the more probable opinion, it may be again inquired, At what point does the fearful complication commence? or what are the first indications of incipient meningitis?"

What then, he adds, are the diagnostic marks of the two diseases, in their earlier stages? Upon the appearance of what symptoms are we justified in assuming the existence of, and adopting the active treatment necessary to subdue, the inflammatory, during the course of the febrile complaint? He conceives that the undoubted difficulty of the answer may be diminished by the following considerations.

In the first stage of acute hydrocephalus, there generally exist some intolerance of light and sound, contracted pupils, and wakefulness by night and by day; while in remittent fever, the patient, though restless at night, often sleeps soundly and comfortably during the day, the pupils are rather dilated, and light and sound are not complained of. The pain of the head in the latter affection is rather a general uneasiness, giving the child an expression of heaviness and languor, and, like the febrile symptoms themselves, is distinctly remittent: in the former, it is almost always referred to the forehead, and, though increased in severe paroxysms, is constant. The child suffering from acute hydrocephalus lays its head heavily on the pillow, with closed eyes, and appears unwilling to be moved, questioned, or noticed; unconsciously moves its hands up to or over its head; and often screams and starts, from severe accessions of pain; while its arms or legs are affected with slight spasmodic twitchings. That affected with the remittent fever, on the other hand, is usually easily and not unwillingly roused: and though fractious and petulent, has not violent fits of screaming; moves its head without inconvenience; and, while awake, is almost always occupied in picking its lips or nose. The bowels are sometimes constipated in both complaints; but they are more easily moved, and, when moved, are more easily kept in a relaxed condition; and the motions are more slimy, fetid, and dark-coloured, in the simply febrile than in the inflammatory complaint. The pulse also, which in the fever is almost always sharp and frequent, is, in the more grave affection, often sluggish, tardy, and irregular.

We fear these diagnostic signs must be taken with some degree of hesitation. But to proceed. Dr. Hughes passes to the complaint first described by Dr. Marshall Hall as a "hydrencephaloid affection of infants," and subsequently noticed by Dr. Abercrombie and by Dr. Gooch. We are tempted, for the matter is a most important one, to subjoin Dr. Hughes's description.

The child is weak, pale, and emaciated; and its flesh loose and flabby: its head is in reality, or from wasting of the face and neck is in appearance, large; it is laid listlessly on its mother's breast or lap, and is raised with difficulty and evident reluctance by the little patient, who is peevish and querulous when disturbed from its languid doze, and soon returns to the position from which it was moved: the features are contracted; the expression faint and distressed; the skin of the face pale, dingy, and wrinkled; it is reported to be constantly disposed to sleep; the pupils are dilated and sluggish: the fontanelles, perhaps, large for the age; the extremities, and general surface, cool; but the head not unfrequently rather hot, especially in occasional flushes; the bowels are relaxed; the evacuations green and slimy; and the stomach often so irritable, as to reject almost every thing introduced into it: the tongue is rather furred, but pale and moist; and the pulse very frequent, and very weak.

In these cases, it will usually be found, upon inquiry, that the child has been ill for some time—rarely for less than a week or ten days,—and that it has previously suffered from symptoms of remittent fever; that it has been prescribed for, by a druggist, or by some medical gentleman who did not see it, and whose view of the nature of the case, and of the remedies necessary to be employed, has been derived from the statement of the mother or nurse; and that these remedies have consisted of leeches to the temples, and purgatives. The symptoms may have previously resembled those of remittent fever; they now are very similar to those of the advanced stages of hydrocephalus. From the previous progress of the disease not having been seen, as well as from the constantly reiterated statement of the mother, that the "complaint lies all in the head,"—to which she perversely adheres, as if for the very purpose of deceiving,—the diagnosis of this affection is often a matter of considerable difficulty.

Dr. Hughes adds, after again admitting the difficulty of accurate diagnosis—This "must be principally derived from, and the treatment essentially founded upon, the history of the case, and the effects of remedies previously administered. For this purpose, the mother must be carefully interrogated, as to the first symptoms, and the progress of the complaint;—whether it commenced with relaxation of the bowels, or indications of inflamed brain;—whether the effects of leeches and purgatives, most probably previously used with incautious freedom, have appeared to be beneficial or deleterious;—and whether the bowels have, or have not, been relaxed, with or without medicine, during the entire course of the disorder. I dwell particularly upon this circumstance, because my own experience leads me entirely to concur in the opinion expressed by Dr. Marshall Hall, that the disease almost universally results from relaxation of the bowels; and because the answers to these inquiries have often relieved my own hesitation, and have directed me to a safe and successful plan of treatment. If then, upon investigation, it be found that the complaint originated in disorder of the bowels—if purging has continued more or less throughout its whole progress, and the head has become only secondarily affected—if, after the application of leeches, the cerebral symptoms have increased rather than diminished, and purgative medicines have aggravated rather than mitigated the disease—I believe, as I have ever found, that stimulants and astringents, with good, wholesome, and nutritious diet, freely and frequently administered, will very speedily remove the worst features of the complaint; and that leeches, purgatives, and mercury, must be immediately and entirely discarded.

It must however be recollected, that venous congestion and serous effusion, with their common indications—somnolence, coma, heavy and even stertorous breathing—may really appear in the last stages of this affection; that they succeed, as the ordinary and perhaps inevitable consequences of extreme exhaustion; and that, with the questionable addition of a blister to the nape of the neck, they can be advantageously and consistently met only by the speedy administration of such measures as are calculated to remove their cause."

### VIII. On the Existence of Arsenic as a Natural Constituent of Human Bones. By G. O. Rees, M.D.

It is well known that M. Orfila has announced the presence of arsenic in human bones and muscles. This is, of course, a most important fact, if it be a fact, and one which would be used with great effect in courts of justice. It is of the first consequence, then, to test its accuracy.

Dr. Rees has carefully repeated M. Orfila's experiments, and added others of his own. They have not corroborated M. Orfila's statements. On the contrary, Dr. Rees has been unable to obtain any arsenic at all. He sums up thus the conclusions which he draws from the experiments in question:—

It appears, that on a careful repetition of the experiments of M. Orfila, with larger quantities of bone-ash; with sulphuric acid, of which the purity was previously ascertained—not by the analysis of a small quantity merely, but by the examination of the whole quantity subsequently employed in the experiment—moreover, by the application both of the test upon which M. Orfila himself places implicit reliance, and of a test which has been shewn to be still more delicate in its indications—with all these precautions, and under circumstances the most favourable for the detection of arsenic had it really existed in bone-ash, a negative result has been obtained.

### IX. Observations on the Absorption of Metals into the Blood, in Cases of Poisoning. Illustrated by an Account of a Case of Poisoning by Lead, occurring in a Cow under the care of Mr. Cherry, Veterinary Surgeon to the Coldstream Guards. By ALFRED S. TAYLOR.

M. Orfila has detected arsenic in the blood of persons poisoned by arsenious acid, both during life and after death—and in the urine and the viscera. Antimony he has detected in the urine and in the substance of the viscera, more particularly the non-secreting organs—and copper has been found in the viscera of *animals* only.

*Lead.*—Mr. Taylor is not aware of any instance of lead-poisoning in which the metal has been found in the blood. A singular case of accidental lead poisoning in an animal, gave Mr. Taylor an opportunity of examining one of the secretions.

Not long since, a cow, belonging to Mr. Harrison, the Treasurer of the Hospital, swallowed a quantity of carbonate of lead, which had been mixed for paint. It appears that some painters had carelessly left the pot containing the paint within reach of the animal. The cow overturned the vessel, and lapped up the contents, amounting, it is supposed, to half-a-pound of dark-green paint. Some time afterwards, the cow was seen, apparently in a state of great suffering, resting, in a contracted posture, with its horns fixed against a wall. There was obstinate constipation; and on the 8th day, general paralysis of the trunk and limbs, so that the animal could scarcely stand. Mr. Cherry exhibited large doses of sulphate of magnesia, and, occasionally, carbonate of ammonia and oil of turpentine; and at the end of a week, Mr. Harrison having applied to Mr. Taylor on the subject, he suggested that large doses of sulphate of soda should be administered, which was readily acquiesced in by Mr. Cherry. This was advised, not so much with the view of the salt acting as a chemical antidote (although commonly employed as such in lead-poisoning), as with the idea of its expelling the poison from the bowels, by its well-known purgative properties. The sulphate of soda was accordingly given in large quantity, dissolved in water; and, apparently, with some good effect. The animal recovered slowly from the action of the poison, and was not perfectly well till the end of ten weeks.

It ought, perhaps, to be mentioned, that at the time of the accident, the cow was rather more than two months gone with calf; and that after three weeks she slunk her calf, which shewed no signs of decomposition, and was of full size for a fœtal calf of that age.

Soon after the animal had taken the poison, and while still labouring under its effects, a quart of milk, drawn from it, was sent to me for chemical examination. It possessed all the properties of rich milk, with an abundance of cream. About an ounce of the milk, deprived of cream, was treated with the hydrosulphuret of ammonia; but this gave no satisfactory indication of lead. Sulphuretted hydrogen gas was then used in its pure state, but without any apparent effect. Judging from these results, that if lead were present in this secretion it could be only in a very minute proportion, Mr. Taylor passed the gas into about ten ounces of the milk; when, after a short time, the whole liquid began to acquire a faint-brown colour. It was allowed to stand some hours; when a black flaky precipitate collected at the bottom of the vessel; but in so small a proportion, that it was impossible to verify its nature by resorting to any of the usual tests for sulphuret of lead. Mr. T. thought it right, however, to perform some comparative experiments on the milk of other cows; and, accordingly, two different specimens were taken, in equal quantities, and treated in a similar way. Sulphuretted hydrogen had no effect whatever in these cases, although the gas was passed in for some hours. On adding a very small portion of a diluted solution of a salt of lead to another quantity of milk, the effect produced by the gas was precisely similar to that witnessed in the case under investigation.

Mr. Taylor adds:—

"These results, affirmative and negative, left no doubt in my mind, that some traces of lead were present in the milk, but in what state it is impossible to say. Admitting its presence in this secretion, it follows, that it must have found its way into the blood by absorption. The quantity present must have been infinitesimally small, since the test was evidently near the limits of its action. The sulphuretted hydrogen gas is said to have the property of indicating the presence of a salt of lead when the latter forms not more than the 300,000th part of a solution. Allowing this to be the case, and judging from a comparative experiment, it appears to me that it cannot be far from the truth to state, that the quantity of the salt of lead contained in the poisoned milk did not exceed the 200,000th part of the weight of the liquid."

### X. Cases of Incised Wound of the Knee-Joint, of Fluid in the Thyroid Body, and of Abnormal Thymus Gland.

1.—*A Case of Incised Wound of the Knee-Joint. By Henry Elston, Esq.*

William Wainwright, of Rainford, aged 45, of muscular make, was, about half-past twelve o'clock on the morning of the 7th of August 1839, proceeding home with a scythe on his shoulder, the blade downwards; and, by some accident, drew it across his knee, and cut deeply into the joint. The knee being flexed, he divided the ligamentum patellæ, and cut off portions of the patella, of the inner condyle of the femur, and of the corresponding condyle of the tibia. These pieces of bone were found on the ground, after he was taken up from the spot where the accident happened, and where he had lain upwards of two hours, on a most tempestuous night. There was very little hæmorrhage, although the wound extended from the inner to the outer condyle of the knee, so that he had nearly amputated the limb. Mr. Elston and Mr. Casey determined, in consultation, very judiciously, to try for union by the first intention. The lips of the wound were, therefore, brought in apposition by the usual sutures, and strips of adhesive plaster, as in the case of an amputated thigh; covering over this dressing with folded linen, wetted in the saturnine lotion. The lotion was continued for a week, to prevent inflammation: suppuration came on about the third or fourth day, but not to any great extent. The limb was, of course, placed in a straight position, and kept so for at least three weeks. No untoward symptom presenting itself, and no treatment being required but the ordinary one in such cases, the patient perfectly recovered, completely regained all the motions of the joint, and was able to follow his usual employment as an active farmer.

This case is possessed of much practical interest. The danger of free wounds into joints has probably been overrated. It is when matter is locked up that they are so *very* formidable.

2.—*Collection of Fluid in the Right Lobe of the Thyroid Body. By J. Massey, Esq.*

A man aged 30, had, for five years, an enlargement of the right lobe of the thyroid gland. He used iodine for three months. In October of last year the following was its and his state:—The tumor is situated over the right side of the thyroid cartilage, extending a little above, and for some inches below it, the longest diameter being obliquely from before backwards, passing under the sterno-mastoid muscle externally. There is no pain on pressure; nor does he complain, except from its inconvenience in size: the measure round the neck, over its most prominent part, is eighteen inches. There is evident fluctuation, which is most apparent at its anterior and inferior surface; here it is more prominent than elsewhere. There is also a pulsation communicated throughout its extent, from its contiguity to the carotid artery. Complexion sallow, and conjunctivæ yellowish—bowels costive—urine high-coloured—wheezing respiration—little cough—vertigo—palpitations—tending to perspirations. Iodine treatment continued till November 27, 1840, when Mr. Massey introduced an exploration needle at the point where the fluctuation was most evident, and evacuated by it about five ounces of fluid, having the appearance of very thin bile, of an olive-brown colour, with an oily crystalline deposit on its surface: this almost completely reduced it to its natural size.

The fluid re-accumulated, when Mr. M. made an opening into the sac, four or five inches in extent, by means of a sharp-pointed bistoury, and evacuated at least five ounces of the same kind of fluid as before: (this was the second fluid, *vide* Analysis; in which was a quantity of blood, escaping from the incision made in the sac and the adjacent parts, which was not in the first quantity, taken Nov. 27). The finger, introduced into the sac, could be passed above the thyroid cartilage, and downwards very nearly to the sternum, and outwards under the sterno-mastoid muscle. The thyroid body was converted into a large cyst, containing the fluid: a pledget of lint was introduced into it, and secured by adhesive straps. A very small quantity of blood was afterwards taken from his arm, for analysis.

The discharge was free, for some time offensive, afterwards purulent. An injection of iodine of the following strength was employed.

Potassæ Hydriodatis ℈i. Iodinii gr. iii. Aquæ distillatæ ℥xvi. m. fiat inject.

A temporary augmentation of the discharge occurred subsequently from cold, but it subsided, and on the 19th Jan. 1841, he is reported as very nearly cured.

The following are the analyses of the fluid as it was withdrawn on the two occasions:—

FIRST PORTION OF FLUID REMOVED NOV. 27.

Slightly Alkaline. Specific Gravity 1.0242.

COMPOSITION.

| | |
|---|---|
| Water | 905.140 |
| Mucus | 19.830 |
| Albumen | 5.210 |
| Gelatine | 11.100 |
| Albumen combined with soda | 8.350 |
| Cholesterine | 10.640 |
| Oily matter | 5.200 |
| Colouring matter soluble in water and alcohol | 8.250 |
| Biliary matter | 9.730 |
| Chlorides of sodium and potassium | 6.210 |
| Carbonates of lime and soda | 4.380 |
| Iron | .250 |
| Loss | 5.710 |
| | 1000.000 |

SECOND PORTION OF FLUID, REMOVED DEC. 4.

Alkaline, and containing Hydrosulphuret of Ammonia. Specific Gravity 1.0356.

COMPOSITION.

| | |
|---|---|
| Water | 896.310 |
| Mucus | 34.270 |
| Albuminate of soda | 7.920 |
| Fibrine | 1.840 |
| Cholesterine | 9.560 |
| Colouring matter soluble in water and alcohol | 16.340 |
| Gelatine | 10.830 |

| | |
|---|---|
| Resin, not affected by nitric or muriatic acids | 5.820 |
| Chlorides of sodium and potassium .. .. | 7.460 |
| Phosphates of lime and soda .. .. | 3.210 |
| Iron, a trace .. .. .. .. .. | |
| Loss .. .. .. .. .. | 6.440 |
| | 1000,000 |

It is probable, as Mr. Massey remarks, that the enlargement of this body primarily consisted in a derangement of its cellular structure, with an increased and vitiated exhalation into the cells, and their subsequent partial or general dilatation and thickening: the previous morbid exciting cause still continuing, an increased secretion into the cells, with their further dilatation and rupture, was the result; so that the lobular structure of this body, in consequence of the additional accumulation, ultimately became converted into a large sac, which contained the adventitious fluid.

### XI. Case of Bone passed from the Urethra.

J. H. aged 45, a patient of Mr. Balderson, of Poland Street, of active habits and irritable temperament, had been subject to stricture in his urethra for a considerable length of time, which was always relieved by the introduction of an instrument. He was in the habit of having a bougie introduced about every three months: but even when performed with the greatest degree of gentleness, he complained of a tightness at the strictured part, and pain upon the instrument entering the bladder. At the beginning of the present year, he called on Mr. Balderson, to have the bougie introduced; and as the inconvenience he always suffered was foreseen, he was strongly recommended to place himself for a few hours, in a recumbent posture: but instead of doing so, he walked as quickly as possible from the neighbourhood of Golden Square to Drury Lane; and having occasion to empty his bladder, he found that more than ordinary exertion was requisite to relieve himself, and a free discharge of venous blood followed. Of this he took no notice: but on the 9th of February, after having attended market, he found that he was compelled every ten minutes to evacuate his bladder; and each time the water was tinged with blood, and a few drops of pure arterial blood followed, producing excruciating pain. He remained in this state during the day; and in the evening sent for Mr. Balderson, who found him with a pallid countenance, a quick and irritable pulse, a coldness of extremities, and he had had shivering. Upon inquiry, it was ascertained that about three weeks previously having slipped off the curb-stone, he had two days afterwards passed blood by the rectum, which he attributed to hæmorrhoids, to which he was occasionally subject. He was sent to bed, and ordered calomel and opium, &c.

He passed a bad night, from frequent desire to make water, with pain in the region of the bladder, which seemed distended with blood. The symptoms increased, in spite of fomentations and opiates, and Mr. Bransby Cooper saw the case in consultation with Mr. Balderson. He prescribed leeches on the perineum and a bladder of ice between the thighs. The patient, however, remained much the same until the 19th, when he shewed Mr. Balderson a substance resembling a fish-bone which he had passed with excessive pain, during the night, through the urethra. The blood gradually ceased, and he was enabled to resume his occupation.

Whether the bone, which caused this patient's complaint, was swallowed with his food, and passed from the rectum to the urethra, or whether it reached that situation in some other way, remains uncertain: that the little substance is really a bone, is evinced by its form, by its cancellated appearance under the microscope, and by its chemical analysis.

### XII. DEFICIENCY OF THE PECTORAL MUSCLES.

A convict, aged 27, was dissected in the Session 1840—1 in Guy's Hospital. Setting aside some other appearances, it was observed that,—

On reflecting the integuments from the anterior parietes of the chest, the ribs, intercostal muscles, and the cavity of the axilla, were at once exposed. The whole of the sternal and costal portions of the pectoralis-major muscle were deficient: but its clavicular origin quite normal: this latter was thick and fleshy, and passed downwards and outwards, to be inserted, by a thin, broad tendon, into the outer edge of the bicipital groove, to the same extent as usual.

The pectoralis-minor muscle was wholly absent; not a vestige of it to be seen.

The serratus-magnus muscle was also, for the most part, deficient, its two superior digitations only being present; arising, as two short, thick, and fleshy heads, from the first and second ribs: these immediately united, and passed to be inserted fleshy into the superior angle of the scapula, in front of the levator scapulæ.

In the left hand, the middle phalanges were absent in all the fingers; except in the middle finger, where a ring of bone, a quarter of an inch in length, supplied its place. The web between the fingers extended to the first phalangean articulation; so that only one phalanx remained free on the distal extremity of each finger. The left thumb was quite normal.

### XIII. ULCERATION OF THE STOMACH—FATAL HÆMORRHAGE.

Mary Waller, aged 49, admitted into Lambeth workhouse Sept. 1, 1836. She had been ill about two months with pain in the epigastrium. About a month previously, she had had an attack of vomiting. She had pain in the region of the heart, the action of which was increased. She was pale and had the aspect of organic disease.

On the 6th, she was suddenly attacked with vomiting of blood, bringing up several clots: this subsided for twenty-four hours, although her power appeared to be very much diminished. The pulse very quick and feeble. On the 7th, a recurrence of the vomiting took place in the evening; when she expired rather suddenly. On the succeeding morning the body was examined. The right auricle was found to be dilated; but otherwise the heart, and large vessels, presented nothing remarkable. The lungs were a good deal congested. The œsophagus was full of coagulated blood; and the stomach distended by two large clots, which were separated by a peculiar constriction of the centre part of the stomach. On opening the stomach, the posterior surface was found adherent to the pancreas; at which part there was a deep ulcer of a sloughy character, with much surrounding hardness. The coats of the stomach itself were destroyed; and the ulceration extended into the substance of the pancreas, penetrating a vessel of considerable size, the open mouth of which was readily discernible, and from which source the blood had evidently escaped.—The pancreas was found rather harder in its structure than usual.

### XIV. CASE OF A REMARKABLE ENLARGEMENT OF THE FEMALE BREAST. By S. ASHWELL, M.D.

Eliz. R. aged 24, short and spare, admitted under the care of Dr. Ashwell,

October 15, 1840, on account of a large tumor of the breast. The following is Dr. Ashwell's note of it upon the 18th.

"The tumor, which is immensely large, measuring twenty-nine inches in circumference, and weighing nearly twenty pounds, is lobulated; the lobes being formed by several almost parallel divisions, running from above downwards. The surface is tolerably smooth, but of much darker colour than other parts of the skin; and branches of veins ramify superficially over it, giving it very much the appearance and tint of the round balls of mottled soap seen in the shops of the perfumers.

The enlargement is uniform, but, although connected with it, does not seem to involve the original structure of the mammary gland; for although what I suppose to be the lactiferous tubes can be distinctly traced from the inner side of the growth into the substance of the gland, still the extent of the healthy mamma can be easily defined. That the lactiferous tubes are enlarged, admits of no doubt; but the great volume of the growth seems to consist in the addition of cellular tissue, containing, I imagine, a large quantity of fat. The whole mass is elastic, and soft; and in no part of it, even on the most careful examination, can I find any trace of indurated or even of slightly-condensed hard structure."

She gave this history:—She began to menstruate soon after twelve years of age: and continued to do so, quite healthily, until her marriage, which occurred at nineteen. She soon became pregnant; and in ten months was delivered of a healthy living child;—the labour having been rather tedious, but in other respects perfectly natural. She suckled with both breasts for the first two months; but was then compelled, in consequence of a diseased state of the nipples, to wean the child. About a fortnight after the accouchement, an abscess formed in the right breast (the seat of the present disease); and in a month discharged itself by a number of small openings, rendering the under part of the gland quite cribriform: these, however, soon closed; and the matter, again collecting, was evacuated by the natural process, through a large opening at the upper part of the breast. The drain consequent on this abscess, and the irritation produced by an attack of erythema nodosum, rendered the weaning absolutely essential.

Within eight months of her first confinement (both breasts being at that time of their natural dimensions and appearance) she again became pregnant. About the twelfth week she perceived a hard swelling, of the size of a hen's egg, in the right axilla: this was neither painful nor tender; but as it increased in size, she applied, under medical advice, some leeches and evaporating washes;—it having been suggested that it was an enlargement of a gland, from irritation. Instead of diminishing under this treatment, the swelling increased; and there was also considerable irritation and pain. Iodine, from her own statement, was subsequently used; but as no better result ensued, it was determined to adopt no further treatment till after her confinement. From this time onwards, to her delivery, which was perfectly natural, the tumor grew: and it measured, on the third day after parturition, eighteen inches in circumference. On this day, the milk being nearly at its height, the tumor, having previously induced inconvenience solely by its size and weight, became exceedingly painful and tense; the uterus appearing to sympathize—as pressure on either could scarcely be borne. Mrs. K. suckled on the left side only; and although the milk still continued to rush into the tumor, it began from this period sensibly to diminish in size, till, at the eighth month, when she weaned on account of pregnancy having again occurred, the morbid growth was not larger than a small orange.

At the end of the third month, as in the second pregnancy, the tumor began again to enlarge, but more rapidly, though without pain. Soon afterwards she went into one of the hospitals; the tumor then measuring twenty-three inches in circumference. From the treatment pursued, Mrs. K. derived no benefit: and she consulted Mr. Callaway, by whom she was sent to Sir Astley Cooper.

After a careful examination, the tumor was punctured with a lancet; and two ounces of curded milk, streaked with blood, were discharged.

The opening soon healed; but shortly afterwards, a collection of fetid matter, intermediate between pus and milk, was evacuated. Within a month of this period, while making some undue exertion, there escaped from the same puncture a large quantity of blood, in a full stream. It stopped spontaneously; but a similar loss occurring shortly afterwards, induced premature labour, at the seventh month. The child was born alive, but survived only twenty hours. Both mammæ were now painful, the right more than the left; and especially the tumor, which, as formerly, having been very tense and hard, began on the third day to diminish in size. The diminution, however, was very different to what had taken place at the expiration of her second pregnancy, when the growth did uot exceed the size of a small orange;—as at the end of six months (May 1840), when she became pregnant for the fourth time, the tumor was still more than fourteen inches in circumference. Now, Oct. 15th, 1840, it measures twenty-nine inches, and weighs nearly twenty pounds.

We need not pursue the details of the treatment adopted in the hospital. It was of no service. She left the institution. She had a natural and easy confinement, and the report on March 17th, four weeks afterwards, is—the tumor has decreased five inches; its greatest circumference being twenty-five, instead of thirty inches and a half as after delivery. The mass is still soft and elastic. Its diminution in size is, however, very slow, compared with what occurred in previous confinements. There is still an opening low down on the external border, which discharges a few drops of cream-like fluid. She says that any undue exertion, such as cleaning her room, is followed by the increase of the growth to about an inch. She seems delicate and feeble; and greatly complains of the burthen of the enlarged breast. There is but little secretion from the right mamma, to which the child is never applied; but on squeezing, a little milk exudes from the nipple, between which and the milk from the sound mamma there is no perceptible difference.

Dr. Ashwell remarks—

"I am not aware—although such have probably occurred—of any recorded cases exactly similar to the one now related. Examples of hypertrophy of the mamma, even of great magnitude, are rare: but several might be adduced, where enormous development has taken place in connexion with, or subsequent to, puberty: but so far as my inquiries have yet gone, I cannot find any instance where the hypertrophy has been so dependent for its increase and decline on pregnancy and lactation. The almost entire disappearance or removal of the growth after the second confinement and suckling, and its return and more permanent continuance after the subsequent deliveries, are points of great interest. Removing the malady, as they appear to do, from the class of malignant diseases, there is yet enough left, about such a case, of a decidedly morbid character, as must excite apprehension for the ultimate result. The diagnosis may not involve any great difficulty, but the probable course and termination of such a disease is far more serious. It is evident, however, much good may be done by light, nutritious, and unstimulating diet, pure country or sea air, cheerful society, and healthful exercise;—that the safety of the patient mainly depends on a quiet inactive condition of the reproductive organs. To what extent, if at all, sexual intercourse, without pregnancy and lactation, may induce the return and the continuance of the disease, it is at present impossible to say; but I believe that the suspension of such excitement would be most important; nor can there be any doubt, when the tumor is permanently developed, that any remedial measures will be useless, so long as pregnancy and suckling continue to recur. An attentive perusal of Mrs. K.'s history will confirm these views. At first, her health was tolerably good; and this unusual result of conception nearly disappeared, after the cessation of the functions which seem to have given it birth.

A repetition, however, of the same actions reproduces, and permanently establishes, the morbid growth: and it is, I fear, but too evident, if this faculty continues in full activity, that fatal consequences must ensue. Her health is already much broken; her nervous system is unduly excited; and her emaciation is marked and progressive. It is not difficult to foresee the result; phthisis, or dropsy of some of the great cavities, will probably occur; or perhaps, previously to the complete establishment of these diseases, the patient may be worn out by the mechanical and constitutional derangements attendant on the malady."

### XV. Observations on the Laws which regulate the Deposition of Tubercles; with Practical Inferences applicable to the Prophylactic Treatment of Phthisis. By George H. Barlow, M.A. & L.M.

Dr. Barlow observes—"In our inquires into the laws which regulate the establishment and progress of tuberculous disease, two questions of great interest meet us, as it were *in limine*; namely, (1) What are the circumstances that induce that constitutional liability to this disease which has been termed the tuberculous diathesis, or tuberculous cachexia? and (2) What are the topical conditions most favourable to the development of tubercles in any particular organ? It is to the latter of these questions that I now offer a reply: and it is this—*That any organ is most liable to become the seat of tuberculous deposit when its vascular and functional activity bears the greatest ratio to that of the other organs of the body.* This conclusion, I say, accords with my own observations; and is confirmed by the facts recorded by many of our most distinguished pathologists, although their opinions have, in some instances, been opposed to it."

After some observations on tubercles in other organs, Dr. Barlow passes to the lungs. Proofs of the truth of the law he has laid down are to be found, he thinks—First, in the period when hereditary tuberculous disease generally first invades the lungs: 2dly, in the external circumstances which are known to be most favourable, or most opposed, to its progress: 3dly, in the part of the lungs which it most affects: and, lastly, in several pathological conditions which appear to influence the development of pulmonary phthisis.

1. Dr. Barlow argues that in childhood, when the respiratory functions begin to be active, tubercles begin to shew a tendency to development in the lungs. This is a time for caution, therefore, and for prophylactic treatment. He next speaks of the period of life when the body has attained its full proportions, or rather ought to have attained them. Then it is that the extremities, and those parts which return their blood directly into the right auricle, have acquired their greatest proportion to those which return it through the medium of the portal system: then it is that the circulation is often accelerated by continued bodily exertion and mental emotion; then too it is, that females most sedulously compress the lower lobes of the lungs, thereby calling into greater activity those portions which are most liable to tuberculous deposit; and then it is that pulmonary phthisis, which perhaps has had its commencement in an earlier period, commits the greatest ravages.

2. *Climate.*—The gist of Dr. Barlow's reasonings on this head is this—that increase of temperature, especially if combined with a humid atmosphere, must increase the action of the liver, and diminish that of the lungs; but that, if the air be drier, the lungs are still relieved, but a greater amount of depurative action is performed conjointly by the liver and the skin: and, conversely, that a depression of temperature must call the lungs into greater activity, by diminishing the functions of the former organs. It also follows, that, since in a warm climate the development of the liver must have been favoured at the expense of that of the lungs, a change to a cold climate must call the latter into a state of over-activity; whilst the same effect must frequently occur amongst those who reside in climates subject to sudden variations of temperature.

And this is precisely what we observe in respect to phthisis: the inhabitants of cold climates are, *cæteris paribus*, far more liable to pulmonary phthisis than those of warm climates; whilst a climate subject to sudden changes of temperature seems still further to favour the production of this disease: and a removal from a warm to a cold climate is frequently found to produce it, not only in men, but also in the lower animals; as is seen in those brought from southern latitudes to the menageries of this country.

It follows, then, that those climates, and those changes of climate, which excite the lungs to the greatest functional activity are precisely those in which phthisis is most readily and frequently produced.

3. *Employment.*—It is notorious, says Dr. Barlow, that those whose occupations expose their lungs to irritating causes are more liable than others to pulmonary consumption; as is seen in those who work in an atmosphere loaded with dust, or charged with irritating gases; whilst a remarkable immunity from this disease appears to prevail amongst butchers, tanners, soap-boilers, and others exposed to exhalations from animal matter. Dr. Barlow accounts for the immunity of the latter class of persons, by the fact of the co-adjutive action of the great depurating organs of the body. We shall readily perceive that the azotized and hydrogenous exhalations to which they are exposed must, if absorbed into the system, be removed by those organs whose function it is to eliminate the excess of such substances, namely, the liver and the kidneys; and, as whatever is prone to pass out of the system by any particular organ, acts as a stimulant to that organ; the liver and kidneys will, in such cases, be excited, and consequently the functional activity of the lungs impaired.

"There is one class of workmen whose diseases I have had great opportunities of witnessing, and amongst whom I have observed an extreme liability to phthisis; namely, hatters. And here, again, we observe an illustration of the same law; for these persons are employed, for the most part, in rooms heated to a very high temperature, and filled with aqueous vapour: under these circumstances, the liver must be highly stimulated; which continued stimulus must give rise to a torpidity, under ordinary circumstances; so that, when they leave their heated rooms, a suppression of the action of this organ, and a corresponding increase of action of the lungs, must ensue. These persons are, in fact, placed in the same circumstances as those liable to sudden atmospheric changes, but in a greater degree."

4. *Part of the Lung most affected.*—The upper part of the lung is, for several reasons, most worked, and is most liable to tubercles.

5. *Pathological Considerations.*—Pregnancy, which draws the blood to the uterus, suspends phthisis.

"Again, a still more unexceptionable proof is afforded in the well-known fact of the arrest of tuberculous disease in the lungs, and consequent prolongation of life, in those cases where pneumo-thorax has occurred from perforation. In these cases, the compressed portion of lung has sometimes been found to have been the only part which is exempt from tubercles. Thus, in a case recorded by myself in the Fourth Volume of this work, perforation of the superior lobe of the left lung occurred; the consequence of which was, that the inferior lobe of the same lung collapsed; and when death ensued, at the end of three years, the portion of lung was found firmly bound down to the bodies of the vertebræ by adventitious membrane, and was entirely free from tubercles; although the remainder of the lungs were so extensively affected by them, that the proper pulmonary tissue was almost entirely obliterated throughout; and the patient died from want of respirable lung, which is rarely the case in phthisis."

Dr. B. draws an additional argument, from the fact, that pulmonary phthisis frequently follows closely upon the suppression of suppurative discharges, as after the removal of a strumous limb; whilst a fresh action in a remote part of the system, whether excited by disease, by accident, or by design, appears to check its progress. Thus, in examining the bodies of those who have died of suppurative affections of remote parts, we often find traces of antecedent or inactive tuberculous disease in the lungs.

6. *Practical Deductions.*—Dr. Barlow concludes:—"The great principle which, in a therapeutical point of view, it seems to establish, is the importance, in the prophylactic treatment of phthisis, of keeping all the excreting organs in a state of moderate activity, and preventing an excessive performance of function by any. When, however, there appears reason to apprehend that undue excitement of the lungs exists, we must endeavour to relieve the functional excitement through the medium of the other excreting organs; and the hyperæmia, by moderate local depletion, and the use of remedies likely to control the action of the heart. The means of fulfilling these indications will readily suggest themselves to the experienced and reflecting practitioner: but as my great object in the present essay has been to establish a pathological principle, though one immediately applicable to therapeutics, I shall not now enter further into details, than to state, that in cases where I have observed a diminished resonance on percussion under the clavicle, with preternatural resonance of the voice in the same situation, occurring in connection with constitutional symptoms calculated to excite the apprehension of phthisis, especially when they presented themselves in a member of a strumous family, I have generally been in the habit of applying leeches or cupping-glasses, and afterwards occasional counter-irritants, and of exhibiting internally sulphate of quinine in combination with digitalis, and some narcotic, which seems most applicable to the particular instance. So large a proportion of these cases has been benefited, that I am much inclined to think that in some the invasion of phthisis has been warded off. The object of the quinine has been to counteract that general debility of the system which seems associated with the tuberculous cachexia; and that of the digitalis to prevent hyperæmia of the lungs, by moderating the action of the heart."

### XVI. History of the Last Illness of Sir Astley P. Cooper, Bart. and Examination of the Body after Death.

For many months previous to his last illness, Sir Astley Cooper had occasionally experienced great dyspnœa, upon the slightest exertion; and it had been observed by his friends, that the peculiarity of his complexion bespoke some serious impediment to the circulation, It was not, however, till about six weeks before his death that he found difficulty in assuming the recumbent posture; and about that time he began to pass the greater part of his nights in the arm-chair, rather than attempt to lie down. He still continued to see a few patients during the day, both at home and at their own houses. He now became the subject of frequent cough; which was immediately brought on, if he attempted to recline. The gout, of which he had for several years experienced periodical attacks, shewed itself imperfectly in the fore-finger of the left hand: and his legs began to swell, owing to the depending position in which they constantly remained.

During all this time he refused medical aid; and it was not till the 22d of

January that he consented to see any one, to whom he might state his symptoms. At the time he was first visited, he was sitting in his chair, with his body inclined forward, and his chin nearly resting on his chest; the pulse accelerated; not the slightest *bruit* nor abnormal sound in the heart; though the beat was extensive, and heard quite to the right side of the chest. The lungs afforded considerable bronchial rattle, but were neither consolidated nor compressed, and filled both cavities of the chest.

Although remedies appeared more than once to produce a temporary remission of his symptoms, and a further attack of gout in one foot seemed to afford some relief to the chest, yet, upon the whole, the disease advanced, and was attended by frightful fits of dyspnœa, during which his face was purple and his mind confused; and it was in one of these paroxsyms that he died, on the morning of the 12th of February.

Shortly before his death, Sir Astley Cooper expressed a wish that the appearances which should be presented on the inspection of his body might be recorded in the Guy's Hospital Reports. He had particularly alluded to four points, the investigation of which he thought desirable;—a cured oblique inguinal hernia; a cured umbilical hernia; some suspected indications of phthisis in his youth; and an inability to sleep whilst lying on his left side.

*Examination of the Body.*—We shall omit what particulars seem unimportant.

There was general and extensive œdema of the lower extremities, and in them only.

The head was not examined.

A globular projection, about the size of a large nut, was found at the umbilicus; which receded on pressure, leaving a well-defined rounded aperture in the linea alba, capable of admitting the end of the little finger. This protrusion consisted of a few congregated lobes of fat placed immediately behind the umbilicus, between it and the peritoneum; the free surface of which was corrugated, and presented a puckered appearance, most probably inflammatory, and the result of the artificial curative means which had been employed for a long period during life.*

The greater omentum, loaded with adipose matter, was contracted, and did not extend downwards more than two inches from the transverse colon. Some very old membranous adhesions existed between the right angle of the colon and the gall-bladder.

Some parts of the surface of the liver were slightly contracted and uneven; and sections of it presented hepatic venous congestion, approaching what is termed a "nutmeg appearance."

The kidneys were surrounded by a considerable quantity of adipose tissue, remarkably dense, and very firmly adherent to the fibrous capsule of the gland. Both kidneys were much congested with blood, rather larger than natural, their surfaces mottled, and slightly granular. These morbid conditions were most evident at the lower part of the left kidney; less advanced, but more generally diffused, in the right: and on the anterior surface of the latter, near its convex edge, were found two small cysts, containing a straw-coloured fluid.

The internal abdominal ring, on the left side, was rendered distinct by a tubular extension of the peritoneum for about an inch into the inguinal canal.

A depression existed in a corresponding situation on the right side, the bottom of which was firm, irregular, and corrugated; and upon very careful examination, a minute serous canal, not more than a line in breadth when opened, was

* Sir Astley wore a piece of cork, adapted to the umbilicus; and maintained in its place by straps of adhesive plaster, during many years, and until his fatal illness.

traced, extending from it, along the spermatic cord, into the cavity of the tunica vaginalis, being the remains of a congenital inguinal hernia.*

Upon raising the sternum and cartilages of the ribs, both lungs were brought into view; and retained their expanded condition, overlapping the pericardium, and manifesting no disposition to collapse. No pleuritic adhesions existed on either side of the chest; nor was there any effusion, except into the right pleural cavity, which contained about three ounces of sanguinolent rather turbid serum.

A little recent pleuritis was found on the middle lobe of the right lung, rendering it slightly adherent, by plastic effusion, to the adjoining lobes, to a small extent. Both lungs presented general vesicular emphysema to a very great degree, and their edges were more rounded than natural.

The larynx was not examined.

The lining membrane of the trachea and larger bronchi was smooth, but of a dark purple hue, from congestion in the minute blood-vessels: the same appearances extended throughout the bronchial ramifications, the smaller of which were filled with a very tenacious puriform mucus; and many of them were observed much dilated. Both lungs were extremely congested with dark blood, especially in and near the central portions of their lobes. At the superior and posterior part of the right lung was a small depressed and somewhat contracted surface, about the extent of a sixpence; a section of which exposed a calcareous mass, very uneven upon its surface, and about equal to the size of a small pea: it was placed about three lines distant from the pleura.

When the pericardium was opened, the heart was seen, very large, and distended; and about two ounces of rather dark or brown-coloured slightly turbid serum occupied the posterior part of the cavity.

The right auricle and ventricle filled with very dark-coloured imperfectly-coagulated blood. The auriculo-ventricular valves sound. Through one of the pulmonary valves, near its angle of union with an adjoining valve, was a perforation nearly the size of a small goose-quill. A tolerably firm fibrinous coagulum was found in the pulmonary-artery and its branches, extending, by minute prolongations, to the fifth divisions: these were made evident, by withdrawing them in a continuous mass with the forceps.

The left auricle and ventricle were occupied by a large quantity of black grumous half-liquid blood. A large portion of the mitral valve opaque, and a little thickened; otherwise healthy. The aortic valves thickened, and rather rigid at their attached margins; whilst the free margins presented a remarkably healthy appearance for their age.

The left ventricle was much dilated; its apex much broader, and more prolonged than natural; the parietes somewhat hypertrophied; and the muscular fibres of the whole organ were pale, flabby, and weak.

The aorta, which was small and narrow, pursued its usual course, but gave off the left vertebral artery between the left common carotid and left subclavian. The entrance to the arteria innominata was contracted, and slightly irregular.

Many small irregular yellowish opaque patches were seen under the lining membrane of the thoracic aorta and the ascending portion of the left subclavian artery. In most of the parts so affected, the internal membrane was much softened, breaking down under slight pressure; at three or four points it was destroyed to a small extent, admitting a thin layer of dark matter, probably altered blood, separating it in a slight degree from the subjacent tissue: this latter state was noticed near the origin of the arteria innominata and the commencement of

* Sir Astley Cooper wore a truss on the right inguinal canal, from the age of 19 to 25.

the descending aorta. The whole length of the abdominal aorta was full of black grumous blood; its parietes thickened; the lining membrane opaque, and raised by the sub-deposition of hard, almost bony matter.

---

### EDINBURGH NEW TOWN DISPENSARY.

### FEVER IN EDINBURGH.*

The medical officers of this Dispensary have given a statistical account of their practice during the year 1840.

Without going into this Report, which would be uninteresting, we may notice the facts relating to *Fever* in Edinburgh.

The months of January, May, and June, show the greatest number of fever cases. Without including febricula and infantile fever, there have occurred, during the whole year, 297 cases of typhus, being in the proportion to the whole practice of 1 to 15. Of these, the acting medical officers have sent 75 to the fever wards of the Royal Infirmary, and of the remainder treated in their own houses, there are accurately recorded 15 deaths, and 188 recoveries, that is, the ratio of the former to the latter is less than 1 fatal case in 12. It is to be remembered, however, that fever cases sent to hospital are in general marked by severity of symptoms, or they occur in families suffering from extreme destitution. Regarding the locality of fever, it is found that 32 per cent. are in the New Town, and 68 per cent. in the Old; or, dividing the whole city into three districts, by two lines drawn from north to south; one through Pitt Street, Dundas Street, the Mound, and George IV's Bridge; the other by Broughton Road, Elder Street, Leith Wynd, and St. Mary's Wynd, the result is, that in the middle one of these divisions, 39 per cent. of fever occurred; in the east division the proportion is as high as 42 per cent., but in the west it is only 19.

These three divisions are not thought to be equally populous, but of late years it has been found that the middle one supplies the New Town Dispensary with the greatest number of cases of all other diseases. The low number of fever cases in the west division, which includes the densely populated Grassmarket and neighbourhood, is probably peculiar to the year 1840; and including their experience during several years, the medical officers are not prepared to select any particular locality in the Old Town as specially subject to typhus. In this respect their views confirm those of Dr. Alison, lately published in the Journal of the Statistical Society of London.

The number of fever cases would seem, if calculations are to be relied on, to amount, in Edinburgh and Leith, to 20,425, or thereabouts.

---

### PERTH INFIRMARY.

### CASES OF HÆMORRHAGE AFTER OPERATION, WHICH OCCURRED IN THE PERTH INFIRMARY. By FRASER THOMSON, M.D., Surgeon to the Infirmary.†

The following cases, indeed all, of secondary hæmorrhage, are interesting:—

CASE I.—*Amputation at the shoulder joint—hæmorrhage—recovery.*—George

---

* Edinburgh Monthly Journal, May, 1841. † Ibid.

Marshall, aged 34, a discharged soldier, admitted July 8, 1840, for disease of the right humerus, near to the deltoid muscle, which has divided the bone, and communicates externally by a large ulceration, discharging an unhealthy matter. The year previous, he was admitted into the house for a malignant disease of the left testicle, which was removed. After consultation, it was determined to remove the arm at the shoulder joint, as there was not sufficient space otherwise to form a good stump. The operation was accordingly performed the next day; five arteries required the ligature; and the stump was dressed in the evening, after all oozing of blood had ceased. For the next two days he did very well; but on the beginning of the third, the stump became suddenly greatly distended, threatening to burst, and arterial blood flowing from the inferior angle. Pressure was immediately made on the subclavian artery with the finger, and maintained for some time, whilst broad straps of adhesive plaster, were applied to the stump to give it support, and the parts kept diligently wet with a frigorific mixture. These means were happily sufficient, and the patient was dismissed cured on the 10th of October following.

Case II.—*Fistula in ano—operation—hæmorrhage—actual cautery—recovery.*—John Grant, weaver, aged 30, admitted 14th February, 1840, for a fistula in ano of seven years' standing. On examination, three sinuses were found, having one common opening at the right side of the anus—one passing backwards, another forwards, and the third upwards, parallel with the gut, but not communicating with it—each about two and a half inches in extent. The two first were laid open a few days after admission, and the third was cut in the usual manner after they had healed. Its walls were found exceedingly hard, almost cartilaginous, having been twice operated on before. The bistoury broke in consequence, but another being at hand, the operation was quickly finished. Little blood was lost, the parts being dressed with oiled lint; the patient was put to bed, and, three hours after, Dr. Thomson was called to him. He had lost about 2 lbs. of blood; and the house surgeon had attempted to arrest the hæmorrhage, both by pressure and styptics, but in vain. A speculum was immediately introduced into the rectum, when the blood was seen flowing freely from numerous orifices. All attempts to apply a ligature having failed, the actual cautery was prepared. A bent copper spatula was introduced to protect that part of the gut not covered by the speculum; the fistula was thus the only part exposed. Every bleeding point was then touched by a heated iron wire, about a quarter of an inch in diameter, and not until this was several times repeated did the blood cease to flow. The patient, as might be expected, was a good deal exhausted, but, nevertheless, made an excellent recovery, and was dismissed cured in the end of April.

Case III.—*Disease of the knee joint—amputation—hæmorrhage—ligature of the femoral twice—recurrence of the hæmorrhage—ligature of external iliac—recovery.*—Thos. Dow, labourer, aged 46, admitted Jan. 17, 1840, with disease of the left knee-joint. An abscess was opened above the articulation, and his health began to suffer. Amputation was accordingly performed on the 24th of May, by the double flap operation—ten vessels required ligature, and a vein near to the bone continuing to bleed very profusely, it became absolutely necessary to tie it also. A large quantity of blood was lost. The patient was in consequence exceedingly faint, but rallied after the administration of stimulants. The stump was dressed in the evening. The progress of the case for the next ten days was very satisfactory, notwithstanding a slight oozing of arterial blood, which was easily arrested by cold and pressure. On the 12th day after operation, all the ligatures had come off, the femoral being the last. On the following day arterial hæmorrhage occurred with great violence, distending the stump and soaking the dressings. The tourniquet was applied to the femoral artery for a few minutes till the vessel was tied. The ligature was passed round it rather less than an inch below Poupart's ligament, in order to be above the origin of the profunda. The bleeding ceased, and cold cloths were applied. The next day, however, early in the morning, it again returned as profusely as ever. The lips of the wound were opened, and the artery secured *immediately below* Poupart's ligament, with very little disturbance to the surrounding parts: the hæmorrhage again ceased, and the cold cloths were continued.

He was now so feeble as to require constant stimulants. He rallied. "On the morning of the 5th day, however, hæmorrhage occurred for the third time. On this occasion the blood flowed from the femoral artery, where it was last tied. The patient himself was the first to detect it, and stopped any further loss of blood, by applying his finger over the vessel; only a few ounces escaped. On going to the hospital, I was fortunate in getting the valuable assistance and advice of my friend Dr. Malcom. After examination, it was evident that the iliac artery must be tied, with what chance of success was by no means certain, from the great tendency to arterial ulceration. The operation was begun after the method of Sir Astley Cooper,—the skin being cut through, and the edges of the abdominal muscles raised. Neither the peritoneum nor the artery could be found. After a careful dissection through fatty matter and enlarged glands, the vessel was felt, considerably nearer to the pubis than naturally, beating very feebly, and much altered in appearance. An unarmed tenaculum, guided by the forefinger of the left hand, was attempted to be passed under it, but, notwithstanding the greatest care, it gave way. The wound was instantly filled with blood, the further escape of which was prevented by the forefinger of the left hand instantly compressing the vessel against the bone. The situation of the patient appeared now to be almost hopeless. It was agreed, after a short consultation, to attempt a ligature still higher up. To enable me to disengage my left hand, Mr. Frew, one of the dispensary surgeons, slipt his hand over mine, and compressed the artery. I then extended the cutaneous incision, upwards and outwards, for about two and a half inches. The fibres of the external oblique being separated, the internal oblique, transversalis, and fascia, being cut through to the extent of the cutaneous incision, the peritoneum was carefully detached from its cellular connections. At this stage of the operation, the difficulty that occurred in finding the artery in the first instance was fully explained. (A tumor of about the size of a small orange, rather flat, occupied the situation of the iliac artery just as it passes out of the abdomen, pushing the vessel to the pubic side, and by its pressure injuring its coats, whilst at the same time the peritoneum was thrown upwards.) The artery was now easily separated from the vein, and, as it appeared to be quite healthy, a ligature was passed under it about an inch below the origin of the internal, and tied with a single knot. The end of the ligature was brought out at the wound, which was closed by one or two points of suture. This last operation was fortunately successful in this nearly hopeless case, and from this time the patient never had an unfavourable symptom. The abdomen, being tender on pressure, was fomented with decoction of poppies, and a few leeches were required once or twice for a slight pain near to the wound. The ligature of the iliac came off on the 18th day after operation, and the patient was dismissed cured on the 31st of July, two months and four days after amputation of the limb."

These are instructive cases.

### EYE DISPENSARY OF EDINBURGH.

### On the Eradication of Inverted Eyelashes: and on the Preparation of Fine Points of Caustics for Ophthalmic and other Purposes. By James Hunter, M.D., F.R.S.E., Surgeon to the Eye Dispensary of Edinburgh.*

Dr. Hunter applies this method to those cases in which the edges of the lids *retain their natural direction*, and *not to cases of entropium*. He applies an irritant to the hair-bulbs, to destroy their reproductive function. He has found tartar-emetic answer best. The following he has found the best method of proceeding.

1. *To ascertain the presence and position of the inverted eyelashes.*—When they are white coloured, and of extreme delicacy, not exceeding the $\frac{1}{500}$ of an inch in diameter, as is often the case, it is very difficult to distinguish them; the edge of the lid should be brought in contact with the cornea, so that the pupil or iris may form a good contrasting background, and though they may not be visible at once, the surgeon should look from a variety of positions, and alter the direction of the light till he sees them. A convex lens of two inches focus, and not less than an inch in diameter, should be held by an assistant so as to condense the light on the parts, which is a much better plan than using it as a magnifier. If, in order to make the puncture, the edge of the lid is to be removed from the cornea, it is well first of all to blacken the pale and delicate eyelashes with any dark extract, such as belladonna, to render them visible, when contrasted with the white sclerotic coat. 2. *The puncturing of the bulbs.*—This should be done with a lancet, or iris knife, entered close to the base of the inverted hairs, *in the direction of their growth*, to the depth of an eighth of an inch, and moved about a little, so as to widen the bottom of the wound, and cut the bulbs. In doing this, it is sometimes advantageous to stretch the part either with a small hook, or over a convex black horn spatula, such as is usually attached to the German wire speculum. 3. *The inoculation.*—This must be deferred till the bleeding has wholly ceased, when the lid being wiped very dry, the drilled end of a darning needle slightly damped, and dipped in powdered tartrate of antimony, or, what answers far better, the tartar emetic point made in the manner to be afterwards described, is to be inserted in the puncture, and held there for a few seconds. 4. *The evulsion of the inverted eyelashes is the last step.*—They should be seized lengthways close to their roots, and drawn with a slight jerk. When common dissecting forceps are used, they should be held *below* their shoulder, and close to their points, which are apt to separate a little when the fingers are placed higher up. The best forceps are those made without teeth; the holding part being merely rough polished, so as not to cut hairs. When the eyelashes are exquisitely fine, and slip through even the best made forceps, I have found it an infallible plan to damp their points with a saturated solution of shell lac in alcohol, and to grasp the hairs for a second or two before pulling them. A momentary, though sharp pain follows the inoculation, and more or less inflammation; but the latter generally subsides in the course of twenty-four hours, and if the operation has been properly done, it recurs in a day or two, in a subacute form, producing a slight pustulation, which, however, is of very limited extent, causing almost no annoyance, but is sufficient to destroy the functions of the bulbs.

* Ed. Monthly Journ. of Med. Science.

2. *How to prepare fine points of caustics, &c.*—" Eight years ago I was called to a case of profuse bleeding from leech bites on the scrotum; after several things had failed, I tried the well-known plan of inserting the sharp point of a stick of lunar caustic in one or two of the bites, which immediately ceased to bleed, but as they were very numerous, the caustic soon lost its point, and I tried in vain to stanch the others with the blunt end. The case being extremely urgent, and the patient nearly pulseless, there was no time to make a new point in the common way, so I wrapt the nitrate in a bit of paper, crushed it with my heel, and placing some of the fragments on a silver half-franc piece, I held it over the flame of a candle, and then dipt the triangular-pointed flat end of a silver probe, previously roughened on a stone, in the melted salt, which adhered, and on cooling formed a fine coating. This being inserted quite into one or two of the bites, they immediately ceased to bleed; to get another point of caustic on the probe was the work of an instant, and, in this way, a most alarming hæmorrhage (the patient being almost in articulo) was speedily arrested. I can recommend the plan to general practitioners when they meet with similar cases, and it will be found an improvement to heat the flat end of the probe before dipping it, and afterwards to hold it in the blue part of the flame for an instant, as in this way a smoother coating is procured. Since the above incident, I have been in the frequent habit of employing the same method to get a fine point of lunar caustic for various ophthalmic purposes, such as touching small sloughy ulcers of the cornea penetrating to the membrane of the aqueous humor, and in many other cases. I have also in this way got a fine coating of the nitrate on a probe, to be passed into the nasal duct in some cases of fistula lachrymalis; and I conceive it might be found useful in many cases in general surgery, such as sinuses, affections of the nose, ear, &c., where caustic would often be introduced, but for the fear of serious consequences from the breaking of a common stick of it in such situations. The plan just recommended answers equally well for caustic potass, when a fine point or globule of it is required, as for producing eversion of a misplaced eyelash, or for destroying the remains of cysted tumors in the eyelids, or other purposes. The potass may be applied to the roughened surface of any common metal; but for the nitrate of silver, the probes must be of silver or gold, else the salt is rapidly decomposed and the probe destroyed. A fine point of tartar emetic, sulphate of copper, and similar salts, may be got by chipping their crystals, and then fixing the fragment on the end of the probe with sealing wax; but another plan, and the only one, when a long bristle, as it were, of these substances is required, is to take a wire, or the point of an old lancet, and heating it, and giving it a very thin coat of sealing wax, to push it whilst hot amongst the powdered salt. In this way a probe, which is to be introduced into a sinus, or into the nasal duct, may be coated with sulphate of copper or any other metallic salt; and this is the method I employ to prepare the flat points of tartrate of antimony for inoculating the bulbs of inverted eyelashes, using for the purpose a bit of platinum foil shaped like a lancet."

The first person we saw use the nitrate of silver on a probe in this manner was Sir Benjamin Brodie. This was many years ago, and we have since adopted the plan very frequently. We have cured, in this manner, several troublesome sinuses, fistula ani, &c. and have noticed it more than once in this Journal.

### CASE OF TEMPORARY AMAUROSIS OF ONE EYE, FOLLOWING THE EXTRACTION OF A TOOTH. By J. HUNTER, M.D.*

In July, 1838, a lad, aged 17, applied at the Dispensary on account of a dim-

* Ed. Monthly Journ. June, 1841.

ness of sight in the left eye, which had come on rather suddenly and under peculiar circumstances. He stated that the sight of both eyes had been very good till four days previously, when, suffering from toothache, he went in the evening to a druggist to have a carious tooth extracted from the left side of the upper jaw. The operation was easily and dexterously performed, and the pain was not particularly excruciating. At the moment the tooth was loosened from the socket, he perceived a brilliant flash of fire before the left eye, which was followed for some minutes by several fainter ones at short intervals. On going to bed an hour or two after, the flashes of fire reappeared, and continued for about an hour, when they gradually ceased. Next day he found the sight of the left eye very much impaired, and all objects seen with it appeared enveloped in a thick mist. He also observed a sort of luminous coloured ring, whirling round, as it were, in the interior of the eye. This state of matters continued much the same for the next two days, and, on the fourth day, he thought there was a decided improvement.

"On examining the left eye, its pupil appeared a *very* little more contracted than that of the right eye, but its shape was perfectly regular, and the motions of the iris unimpaired. In every other respect, too, the organ presented a perfectly natural appearance. The general health of the lad, who was of a somewhat sanguineo-nervous temperament, was good. He had no pain in the eye or in the brow, and no symptoms of cerebral congestion, or of derangement of the digestive organs. When he closed the right eye, the sight of which was sufficiently acute, and looked with the left one at the page of a book, printed in a type which I have since ascertained can be easily read by a good eye at a distance of 48 inches, I found he could not make it out at a greater distance than about 15 inches, and even then with difficulty, and at any nearer distance, though the letters appeared larger, they still seemed to run into each other. When I tried him with type about one-half the size of the first, he was unable to read a single word of it at any distance." "Neither convex nor concave spectacles improved his sight. His perception of colours appeared somewhat impaired, but I had not the means at hand of examining carefully the condition of the eye in this respect. The tooth which had been extracted was the first great molar on the left side of the upper jaw. On making firm pressure with the points of my finger in its socket, there was no unusual tenderness nor any shooting nervous pain produced, and I could not discover any remaining portion of the tooth, nor any splintering of the alveolar process."

Dr. Hunter left the case to nature, and at the end of a fortnight he could distinguish all but very minute objects. He did not return afterwards, so that his recovery was probably complete.

---

### ROYAL INFIRMARY OF EDINBURGH.

### Case of Urinous Vomiting.*

Dr. Douglas reports this case from the practice of Dr. Henderson.

Marion Purdie, aged 25, a house servant, had laboured, for about six years, under the various pains and aches, accompanied with difficulty of micturition, which young women, out of Scotland, as well as in it, are very prone to suffer from. She was admitted in September, 1840, with pain in the loins and head, she had passed no water for two days previously, and now what she made was dark coloured and scalded her. The catheter was introduced pretty regularly—

* Ed. Monthly Journ. June, 1841.

she had a *hysterical fit*—and on the 14th September she passed 18 oz. of urine, and vomited 8 oz. of a fluid very similar to urine in appearance and odour. The matters vomited were received into a clear vessel, and instantly removed to a place of security.

"My friend Dr. Philip Maclagan, now Staff-Assistant Surgeon at York, then proceeded to analyze the fluid, and found that, after the addition of nitric acid and gradual evaporation, crystals were deposited; these were dissolved in a small quantity of tepid water, and after standing, most of the impurities having subsided, the clear liquor containing the nitrate of urea in solution was poured off and evaporated. The crystals thus obtained were pressed between the folds of bibulous paper, and afterwards dissolved in pure water; this solution was evaporated at a slow heat, when the crystals were again formed. I think it right to state, that I did not myself witness the act of vomiting, but I have no reason to doubt the testimony of the person who was present, and at my request took the precaution to receive the matters vomited into a clear vessel.

In the beginning of October she was attacked by typhus fever, probably from infection. The attack, though of short duration, was a smart one, and was accompanied with the usual eruption. On the second or third day of the fever, she had a fit similar to that formerly described, but less severe. The urine continued in its usual small quantity until the eighth day of the fever, when she was seized with vomiting, and soon afterwards she passed 16 oz. of urine.

The matters vomited presenting a yellow frothy appearance, as if containing a considerable quantity of bile, were carefully preserved; and from them, with the assistance of my friend Dr. Philip Maclagan, I obtained, by the process formerly described, some crystals, undoubtedly consisting of the nitrate of urea."

The patient, after this, steadily convalesced. Dr. Douglas adds :—

"Considering the cases of this kind which have been already published, I would not have brought forward the present one were it not that I have been enabled to avoid those sources of fallacy to which cases formerly published were exposed. The subjects of this disorder, mostly hysterical women, have been known in some cases to mingle urine with the matters vomited; in others, to swallow urine, and then excite vomiting. Besides, I do not know of any case in which the matters vomited were analyzed, their identity with urine being presumed merely upon the similarity of appearance and odor.

In the present case vomiting occurred when the patient was in that condition, so common in the advanced stage of fever, in which she was unable to turn herself in bed; so that the possibility of her having deceived us is put out of the question; and again, the analysis of the matters vomited, places beyond a doubt the existence of urea in them."

And yet we are not sure we believe this. The woman *may* have put some urine in the vessel—*may* have swallowed some—some urine *may* have been accidentally in the utensil on the last occasion (the long interval between the vomitings would have deadened precautions)—Dr. Douglas's informant *may* have been careless—or the same informant *may* have been an accomplice—or one hypothesis *may* explain the first occurrence, another the second—in short, this *may* have been a piece of legerdemain not a bit more extraordinary than some of the animal magnetism flams—and much more likely and credible than a departure from the ordinary laws of nature. For, be it observed, that all these cases occur in young women. From the Okeys downwards, *they* are the favoured agents of the marvellous.

By the way, we observe in the Journal before us another of these queer cases —one of *coma* in a young girl, lasting an immense time—a wonderful *and* a hysterical complaint. There is no end to them.

### OPHTHALMIC HOSPITAL OF CANTON.

It appears that this hospital has existed for some time, and that the natives were beginning to appreciate its benefits. But his pig-headed celestial majesty was unwilling that his subjects should be cured by barbarians, and resolved on an ophthalmia monopoly. The fortunes of the institution have been checquered of late.

It increased in prosperity up to the 23rd of March, when foreigners, one and all, were deprived of their servants, and in a manner of their liberty. At the commencement of this state of things, the few indoor patients were desired by the senior hong-merchant to remove, and the hospital was closed. After a while, however, the officers on guard around the factories began to seek medical aid; and, though they permitted no communication with the people in general, they presently gave admittance to the physician's house, to men of rank, who were allowed a greater measure of liberty in visiting the factories. On the withdrawal of the soldiery and armed coolies, the number of patients gradually increased, but with a greater preponderance than before of official people: yet others were not wholly restrained from seeking relief for their maladies; females even overpassed the prejudices against entering the factories of foreigners. It was in August, that, finding his private residence too small for the reception of the increased number of patients, and unsuccessful in every endeavour to return to the building formerly occupied, the physician removed to the premises of the Canton Dispensary of Messrs. Cox and Anderson.

Commands were issued by the chunghee, against any natives passing in front of the factories, be they men, women, or children: this was applied chiefly to females by the subordinate officers, who were anxious, in consequence, that no females should be received as patients. A few days after, however, appeared a young woman of about sixteen years, from the family of the kwanghee. This officer, of like rank with the chunghee, both being what we may call brigadiers, was associated with him in the control of the foreign factories; and the breach, by one from his own family, of his colleague's orders, reduced these at once to a dead letter. There was no longer any hindrance to the access of females: they came, however, with more of reserve than formerly; and some begged to be seen in boats before the factories, or at their own residences in the suburbs of the city: so that the evil of exclusion from the former hospital has not been without its advantages, inasmuch as it has given rise to a more ready and more frequent access into private families than otherwise would have been thought requisite.—The young woman from the kwanghee's family came with cataract of both eyes; and, though she would not remain as an in-door patient, but returned as soon as the cataracts were operated on, the operation was completely successful.

The smaller number of patients attending, in consequence of various restraints has happily left more leisure for prosecuting the study of the language, and for acquiring facility of writing it, as well as of translating from it.

Among the more distinguished personages who have, directly or indirectly, availed themselves of the benefits that the institution affords, were, Howqua, the senior hong-merchant; Tsun, an officer from Yunnan; Lew, magistrate of Nanhae, and his brother; Wang, a commissioner or intendant of circuit in Kwangse, son of the Van tazhin of Macartney's embassy; the ganchasze, the poochingsze, heads of the judicial and the financial and territorial affairs of this province,—and, not least, the high imperial commissioner Lin, of whom all have heard so much.

The total number of patients that have been admitted and their names recorded, during the year 1839, has been 800: the aggregate number since the commencement of the institution in November of 1835, about 7000.*

---

### STEEVEN'S HOSPITAL.

### A Case of Hæmorrhagic Diathesis. By S. Wilmot, A.B., M.D. One of the Surgeons, &c.

In the year 1837, a boy four years old was taken to Dr. Wilmot's house, in consequence of a bleeding from the palate, just behind the alveolar process of the incisor teeth; a very superficial abrasion of surface could be observed, from which blood continually flowed. The bleeding had existed for four days before Dr. W. saw him; the blood was very pale, watery, and did not coagulate; pulse very quick, and the entire surface of the body was extremely pale. Dr. W. applied the nitrate of silver, used pressure, and prescribed the infusion of roses, with sulphuric acid and tincture of digitalis. The flow of blood did not entirely cease for five days. In about two months a small tumour appeared over the right parietal bone; attempts were made to effect the absorption of this swelling without success; it suppurated broke, and discharged pus. The following day a profuse hæmorrhage took place from the cavity of the abscess, which was arrested by pressure, and no return of bleeding took place after the fourth day. He soon began to gain strength after this attack. While in this state of improvement he fell, while playing, over a bucket, and received a transverse wound of the tongue, which divided the lingual artery on the left side. After the lapse of two days, he was brought into Steeven's Hospital, and the artery tied immediately on his admission. He had lost a large quantity of blood before he came into hospital, but recovered much sooner than could have been expected, and continued in tolerably good health for about seven months after the accident, when he was suddenly seized with bleeding from the gums of the incisor teeth of the upper jaw, which continued for three weeks. He was now readmitted into hospital, and appeared almost exsanguine, and was obliged to be kept in the horizontal position to prevent fainting. The bleeding was obstinate. The treatment adopted in this attack consisted of astringent lotions and pressure applied to the gums; the internal remedies consisted of infusion of roses, sulphate of alum, and tincture of digitalis. On his return home the patient was attacked with fever, accompanied by delirium. After his recovery his parents sent him to the country, where he rapidly improved in health; the recovery was so remarkable that at the expiration of two months he was taken home, all apprehensions of a future attack being removed. The father and mother were, however, sadly disappointed, for he was little more than four months in town when he began to lose his appetite, flesh, and strength. While in this state of declining health he was seized with a pain and swelling in the calf of the left leg. The swelled parts were black and yellow, presenting all the appearance of an ecchymosis from an injury of some days' standing. The mother said that she often observed black spots on different parts of his body which soon disappeared. He was free from hæmorrhage. Dr. W. ordered a cold lotion to be applied, and a mild purgative combined with bitters. The swelling and blackness had scarcely disappeared when he complained of pain in his head, which was succeeded by a bleeding from both nostrils. This attack of epistaxis was of five days' duration before Dr. W. saw him. The mother applied several popular remedies at home, and was induced to postpone seeking medical aid from day to day, owing to occa-

---

* Dublin Journal, May, 1841.

sional cessation of the bleeding for short intervals. The blood coagulated this time though the child was very weak and pale. Plugs of coagulated blood were formed now and then in the anterior nares; when this took place the blood passed into the pharynx and mouth. The epistaxis gradually subsided. The alarm which this poor little afflicted boy suffered after every attack of bleeding had not had time to subside, when a violent pain struck him in the left thigh; a swelling of the whole thigh soon took place. There was now no discoloration of the skin; he could not bear the swollen part to be handled in the gentlest manner, nor could he move that limb in consequence of the pain. He lay with the knee bent and the thigh flexed on the pelvis, and complained of starting and throbbing of the limb. There was a high degree of symptomatic fever. About the third day the pain abated considerably; the fever and swelling were now a good deal reduced; the limb became black and yellow, presenting an ecchymosed appearance. Before he was sufficiently recovered to leave his bed, the thumb and fore-finger of his right hand became painful and swelled, and in two or three days assumed a black and yellow colour. Since this attack, which occurred about the middle of July, he has had no regular medical treatment, and the disease is still unsubdued. He is now afflicted with a diarrhœa, but there is no bloody discharge from the bowels. His mother says that the contents of his bowels flow from him with great force, small quantities of blood come from both nostrils about every ten days. There are a few black spots on the trunk and arms about the size of a silver fourpence. At the last date of Dr. W.'s report, the boy was taking decoction of logwood with alum.

The father and mother are healthy. A brother and two sisters are also healthy. A sister of the mother's lost two boys from hæmorrhage, one, about four months old, died of bleeding from the gums after they were lanced; the other bled to death from an incision into an ecchymosed tumour produced by a fall.

